# Critical ischemia duration thresholds in the rat middle cerebral artery occlusion model: Implications for drug screening

**DOI:** 10.4103/NRR.NRR-D-24-00972

**Published:** 2025-11-25

**Authors:** Alba Puente-Sanz, Amanda Herrero-González, Diego Pérez-Rodríguez, José Manuel Gonzalo-Orden, Michal Letek, Berta Anuncibay-Soto, Arsenio Fernández-López

**Affiliations:** 1 Departamento de Biología celular y molecular, Universidad de León, León, Spain; 2 Neural Therapies S.L., Edificio Institutos de Investigación, León, Spain; 3 Departamento de Medicina, Cirugía y Anatomía Veterinaria, Universidad de León, León, Spain

**Keywords:** behavioral assessment, gene expression, infarct volume, ischemic stroke, middle cerebral artery occlusion, neurological deficit, preclinical model, reperfusion injury, transcriptomic analysis

## Abstract

The growing incidence of stroke and the absence of drugs to fight its devastating consequences highlight the urgent need to identify therapeutic agents. The middle cerebral artery occlusion model is commonly used to screen new putative anti-stroke agents in rodents. However, differences in ischemia and reperfusion times result in differences in neurological deficit and infarct volume, two of the most commonly used parameters in preclinical assays. These differences make it difficult to select the optimal screening conditions. Here, we report a parallel study comparing behavior, infarct volume, and transcriptomic outcomes after 15 days of reperfusion in a rat middle cerebral artery occlusion model with mild (45 minutes) and moderate (60 minutes) ischemia. The behavioral assays revealed that motor and sensory responses, but not the infarct volume, are sensitive to slight variations in the time of ischemia. The transcriptomic profiling demonstrated striking changes in the number of up- and downregulated genes between mild and moderate ischemia. Gene Ontology analysis supported noteworthy differences between mild and moderate ischemia, particularly in genes related to protein synthesis and processing. Complementary quantitative polymerase chain reaction and western blotting assays confirmed these differences. These results indicate a threshold between mild and moderate ischemia, where changes in behavior and molecular responses mirror a substantial alteration in brain cell biology that appears critical for molecular screening. These findings pinpoint the critical role of selecting the appropriate ischemia model in preclinical stroke studies to enhance the translational relevance of the results and therapeutic strategies.

## Graphical abstract

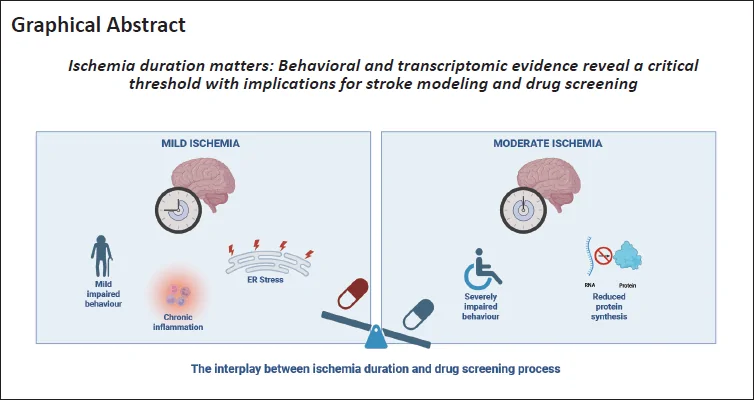

## Introduction

Stroke is considered the second leading cause of mortality throughout the world and the third leading cause of death combined with disability. From 1990 to 2019, the global stroke burden increased by 70% for incident strokes, 102% for prevalent strokes, and 43% for deaths from stroke. Moreover, there was a 14% increase in disability-adjusted life-years (DALYs) (Feigin et al., 2022). Despite the early success of many molecules in preclinical assays against different targets, the only approved pharmacological treatment for stroke is tissue plasminogen activator (Dhir et al., 2020; Ruscu et al., 2024; Xie et al., 2024), which presents significant limitations. Specifically, a short therapeutic window (about 4 hours after the stroke onset) and side effects (hemorrhagic transformation) restrict the use of tissue plasminogen activator in stroke patients (Dewar and Shamy, 2020). The poor success in transitioning from preclinical assays to clinical applications has been presumed to depend on the low quality of preclinical study designs (Friedländer et al., 2017; Ruscu et al., 2024).

In this regard, behavioral assessments are crucial tools for testing putative therapeutic agents against stroke in animal models. Most experimental work has been performed in rodents using the middle cerebral artery occlusion (MCAO) model, which is considered the most relevant to human ischemia (Li and Zhang, 2021; Li et al., 2023; Zeng et al., 2023). However, behavioral analysis presents many limitations because several factors can influence the results, such as the anesthetic drug, the surgical procedure, the environment, and/or circadian rhythmicity (Balkaya et al., 2018). Moreover, rodents present an extraordinary ability to recover motor control 3–4 weeks after focal ischemia, and sensorimotor deficits can only be detected if sensitive tests are employed (Balkaya et al., 2013a, b, 2018). Furthermore, different ischemia and reperfusion times used for MCAO result in dissimilar behavioral outcomes, requiring the use of different behavioral tests to evaluate the neurological deficit. There is little consensus on the most appropriate behavioral tests to assess deficits, especially when testing over extended reperfusion times in relatively mild models that have a short ischemia time and induce a small lesion volume (Trueman et al., 2017). Similarly, infarct volume measured by 2,3,5-triphenyl tetrazolium chloride (TTC) is widely used in drug screening in animal stroke models. However, the reliability of this method, the variability of the final infarct size, and the most suitable time point to measure the effects of drugs that are being screened are controversial, and this parameter should be used with caution (DeVries et al., 2001; Liu et al., 2009; Friedländer et al., 2017).

The most common ischemia times in the rat MCAO model are 60, 90, and 120 minutes, as well as permanent occlusion (Carmichael, 2005). Several studies have demonstrated that 60 minutes of occlusion (in this report, we arbitrarily call this moderate ischemia) results in a consistent infarct volume and neurological deficit in the hours to days following the occlusion (Santos-Galdiano et al., 2018, 2021; Ugidos et al., 2023). Moderate ischemia induces widespread damage in diverse brain regions, resulting in complex motor, sensory, autonomic, and cognitive deficits, making it difficult to analyze stroke progression. Thus, we looked to model milder damage by decreasing the ischemia time, considering that an overall perspective of gene expression changes between mild and moderate ischemia, in parallel to behavioral and infarct volume assays, could improve the reliability of preclinical studies. Previous studies indicate that 30 minutes of occlusion-induced ischemia did not produce brain infarct in normal Sprague–Dawley (Mišir et al., 2016), while 45 minutes of ischemia already led to enhanced astrogliosis in mice (Tachibana et al., 2017). Therefore, a 45-minute occlusion was selected to achieve minimal but detectable brain damage. Here, we present the first study comparing the messenger RNA (mRNA) expression and behavioral responses of mild and moderate ischemia after 15 days of reperfusion. We found striking differences in functionality between the mild and moderate ischemia models, and these differences could play a highly relevant role in the screening of drug candidates.

## Methods

### Animals

Thirty 12-week-old male Sprague–Dawley rats (430–560 g) (Janvier Labs, Le Genest-Saint-Isle, France) were used. This study used only male rats to minimize variability related to hormonal fluctuations associated with the estrous cycle in females, which can influence ischemic outcomes. This approach aimed to ensure consistency in the experimental model and improve reproducibility. This approach has been chosen to continue the experimental assay previously published (Ugidos et al., 2023). Animals were randomly divided into three experimental groups, with 10 animals in each group: sham, 45 minutes of MCAO (mild ischemia), and 60 minutes of MCAO (moderate ischemia). The animals were housed at 22 ± 1°C under a 12-hour photoperiod and with food (Panlab, Barcelona, Spain) and water available *ad libitum*.

**Figure 1:**
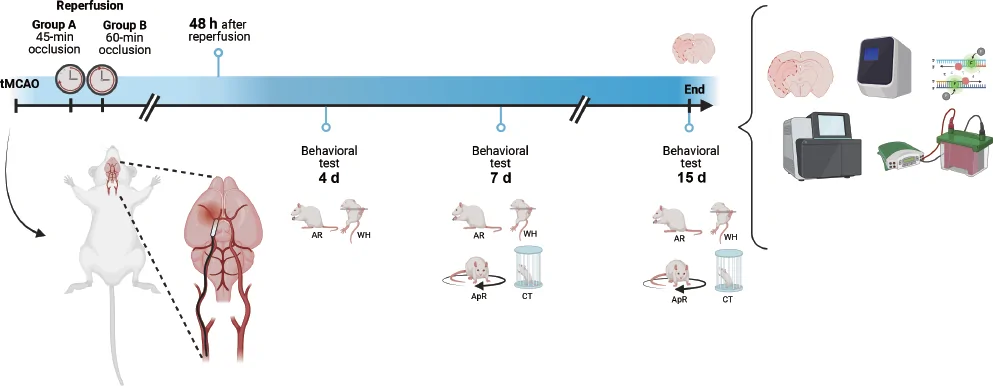
Overview of the experimental workflow. The upper figure shows the experimental groups (A and B) subjected to mild (45 minutes) and moderate (60 minutes) ischemia, the times when the behavioral assays were performed, and the end point at which the samples were collected. The figure at the bottom left shows the MCAO procedure to occlude the middle cerebral artery. The figure at the bottom right indicates the region selected to quantify messenger RNA and protein expression. ApR: Apomorphine-induced rotation test; AR: adhesive removal test; CT: cylinder test; mNSS: modified neuroscore test; tMCAO: transient middle cerebral artery occlusion; WH: wire hanging test.

All efforts were made to minimize the number of animals used and to avoid suffering. All procedures were carried out in accordance with the Guidelines of the European Union Council (63/2010/EU) and the Spanish regulation (RD53/2013) for the use of laboratory animals, following the Animal Research: Reporting of *In Vivo* Experiments (ARRIVE) guidelines. The procedures were approved on June 20, 2022 by the Scientific Committee of the University of Leon (León, Spain) and Junta de Castilla y León (reference OEBA-ULE-006-2022). The experimental workflow overview of the study is shown in **Figure 1**.

### Middle cerebral artery occlusion procedure

The transient MCAO procedure was performed as described previously (Ugidos et al., 2017; Santos-Galdiano et al., 2021). In brief, 3.5%–4% isoflurane (Esteve, Barcelona, Spain) in 100% oxygen-enriched air was used to induce anesthesia with a flow of 2 L/min in an anesthesia box; subsequently, anesthesia was maintained with 2% isoflurane. Each rat was placed on a heating pad at 36 ± 1°C to maintain its body temperature. MCA blood flow was monitored with a Doppler probe (Perimed, Järfallä, Sweden) fixed on the temporal bone with cyanoacrylate. A coated filament (Doccol, Sharon, MA, USA) was passed through the right common carotid artery and then the internal carotid artery until it finally obstructed the MCA origin. At that point, the Doppler laser should show at least 80% blood flow restriction; rats with < 80% blood flow restriction were not used (Ugidos et al., 2023). After 60 minutes (moderate ischemia) or 45 minutes (mild ischemia) of occlusion, depending on the experimental group, blood flow was restored (i.e., reperfusion began) by removing the filament. The reperfusion was confirmed with the Doppler probe. The sham group underwent the same procedure except for MCA occlusion. Due to the high risk of infection when using such long reperfusion times, enrofloxacin (5 mg/kg) (Ecuphar, Oostkamp, Belgium) was used during the first 5 days post-operation.

### Infarct volume measurement

The cerebral infarct volume was measured following the TTC method, the most commonly used method to evaluate brain damage after stroke. This compound acts as a redox marker, detecting metabolically active and non-active tissues. After 2 or 15 days of reperfusion following mild or moderate ischemia, each rat was decapitated and its brain removed and placed in a cold rat brain matrix (ASI Instruments, Warren, MI, USA) to obtain 2-mm-thick coronal sections. These sections were incubated for 15 minutes in 1% TTC (Thermo Fisher Scientific, Waltham, MA, USA) in 50 mM phosphate-buffered saline. Tissue with mitochondrial dysfunction maintains its white color, whereas in tissues with active mitochondria, TTC is enzymatically reduced by mitochondrial dehydrogenases to 1,3,5-triphenyl formazan, which has a deep red color and thus allows identifying active (colored) and non-active areas (Ugidos et al., 2023). Then, the sections were fixed overnight in 4% paraformaldehyde in 50 mM phosphate-buffered saline (pH 7.4) at 4°C and placed on transparent film to be digitized at 600 dpi resolution with a scanner (Canoscan LIDE 200, Canon Inc., Tokyo, Japan). Fiji (National Institutes of Health, Bethesda, MD, USA) was used to estimate the infarct volume by measuring the white, metabolically inactive areas. The results are expressed as a percentage of total brain volume. A correction to adjust edema was made according to the methods of Reglodi et al. (2002). In brief, the infarct volume was calculated as total volume of the ipsilateral hemisphere/total volume of the contralateral hemisphere, and the percentage of the infarct volume was calculated as 100 × (non-stained volume [mm^3^] /edema index)/total volume [mm^3^]).

### Neurological deficit

The behavioral tests were video recorded and subsequently evaluated independently by two blinded researchers. The following tests were performed.

The neurological deficit after 48 hours was evaluated based on the modified Neurological Severity Score (mNSS) (Senda et al., 2011) following Santos-Galdiano (2018). In brief, five tests were performed to evaluate the neurological deficit: (1) postural reflex (hanging test) recording the extension of the leg (scored 0, 1, 2, or 3); (2) circling motion testing if the rat has straight-line locomotion (scored 0, 1, 2, or 3); (3) falling to the contralateral side, recording the magnitude of the fall (scored 0, 1, or 2); (4) placement of the right forelimb during locomotion, recording the use of the contralateral forelimb (scored 0, 1, or 2); and (5) the general state of alertness or consciousness (scored 0, 1, or 2). The total neurological score is obtained by adding the score from each behavioral test; it ranges from 0 (non-injured) to 12 points. For this study, the final neurological score was calculated as the average of the total score obtained by two independent observers.

The cylinder test used to assess the forelimb response was performed at three time points: before surgery and then after 7 and 15 days of reperfusion. A clear plastic cylinder 37.5 cm high and 13 cm in diameter was used to film the number of forelimb contacts with the cylinder surface for 10 minutes. A minimum of 20 contacts was set as an exclusion criterion before MCAO. The results are expressed as the mean of the two observers, and the following formula was applied to calculate the bias on the contralateral forepaw use: 100 × (contralateral contacts)/(ipsilateral contacts + contralateral contacts) (Trueman et al., 2017). Any rat that presented < 35% or > 65% contacts with one paw before surgery was not considered.

The adhesive removal test was used to assess the detection and removal times of an adhesive label placed on the contralateral forelimb at four time points: A square piece of adhesive tape (1 cm × 1 cm) was stuck to the left forepaw pad of the rat. The total latency for removal was measured as the time from the tape being stuck until it was completely removed. This removal is considered to rely on two components: sensory deficits, recorded as the time when the animal detects the tape, and motor deficits, recorded as the time between the rat detects the tape and removes it completely. The procedure was performed according to the methods of Schaar et al. (2010). In brief, three trials for each paw (alternating between left and right) were recorded at each time point. Between trials, the animal was returned to its cage for at least 10 minutes to recover. The results are expressed as the mean of the three trials for each paw at each time point. When a rat was not able to remove the adhesive tape, it received a maximum score of 300. Pre-surgery tests helped to exclude animals that did not behave as expected (taking more than 200 seconds to remove the adhesive tape or showing significant asymmetry between their forepaws in the time taken to remove the adhesive).

The wire hanging test was performed to assess neuromuscular strength and motor coordination at four time points according to the methods modified by Owjfard et al. (2024): before surgery, and after 4, 7, and 15 days of reperfusion. A semi-transparent red plastic cube 80 cm high and 65 cm wide, with a wire placed end-to-end at the top, was used for this test. The bottom of the bucket was cushioned to avoid injuring the rat. The time the animal stood hanging on its two forepaws was recorded (three trials per time point). Between trials, the rat was returned to its home cage for at least 10 minutes to rest. The total score is expressed as the mean of three trials at each time point.

The apomorphine-induced rotation test was performed to evaluate dopaminergic asymmetry and motor function at two time points according to the methods modified by Trueman et al. (2017): after 7 and 15 days of reperfusion. Apomorphine (1 mg/kg; Lot# SLCB4167, Sigma-Aldrich, St. Louis, MO, USA) was administered subcutaneously, and the number and direction of the turns after the injection were counted over 30 minutes (starting from 30 minutes after the injection). The results are presented as the changes in turning rate over time to determine ipsilateral bias (ipsilateral/right/clockwise turns – contralateral/left/counterclockwise turns). The total number of turns was also measured. In both cases, the data are expressed as the number of rotations per minute.

### RNA analysis

After 15 days of reperfusion, the brains were removed and sectioned with a brain matrix (Rodent Brain Matrix, ASI Instruments) to obtain 2-mm-thick coronal slices from bregma –0.8 to bregma 1.2 (Paxinos and Watson, 1998). The primary somatosensory forelimb area (S1FL) was dissected from bregma 0.8 of the ischemic hemisphere and stored at –80°C. The TriPure isolation reagent (Roche, Basel, Switzerland) was used to extract protein for western blotting and total RNA from frozen tissues following the manufacturer’s instructions.

### Transcriptomic analysis

mRNA from the S1FL ischemic brain hemisphere was purified using oligo(dT) magnetic beads (Novogene, Beijing, China). After fragmentation, first-strand complementary DNA (cDNA) was synthesized using random hexamer primers, and then second-strand cDNA was synthesized using either dUTP for directional library or dTTP for non-directional library (Parkhomchuk et al., 2009).

The non-directional library was ready after end repair, A-tailing, adapter ligation, size selection, amplification, and purification. The directional library was ready after end repair, A-tailing, adapter ligation, size selection, USER enzyme digestion, amplification, and purification. The libraries obtained were quantified with a Qubit fluorometer (Thermo Fisher Scientific) and quantitative reverse transcription-polymerase chain reaction (qRT-PCR), and a bioanalyzer determined the size distribution. The quantified libraries were pooled and sequenced on an Illumina platform according to the effective library concentration and amount of data (Novogene).

### Quantitative reverse transcription-polymerase chain reaction

The RNA sequencing (RNA-seq) results were validated with qPCR, carried out in accordance with the Minimum Information for Publication of Quantitative Real-Time PCR Experiments (MIQE) guidelines (Taylor et al., 2010). After Tripure isolation, RNA was subjected to quality control analysis and quantified using a Nanodrop (Thermo Fisher Scientific). The integrity of the isolated RNA was checked by running it in a 2% agarose gel. Samples with a 280/260 ratio of < 2.0 or without two clear bands (corresponding to ribosomal RNA [rRNA]) after electrophoresis were discarded. cDNA from samples meeting the requirements was obtained using a Reverse Transcription Kit (Applied Biosystems, Bedford, MA, USA). The primers used for qPCR were designed using primer-BLAST from NCBI (see **Table 1** for the sequences). Each reaction comprised SYBR Green PCR Master Mix (Applied Biosystems), 1 μL of 10-fold dilution of cDNA (600 ng), and 300 nM primers. The reactions were run on a StepOnePlus^TM^ Real-Time PCR System (Applied Biosystems) under the following conditions: 2 minutes at 50°C, 10 minutes at 90°C, and 40 cycles of 15 seconds at 95°C and 1 minute at 60°C. Primer specificities were checked by melting curves and agarose electrophoresis analyses. Primer efficiencies were determined by using increasing cDNA template concentrations (**Table 1**). Differences in transcript levels were analyzed using the 2^–∆∆Ct^ method (Livak and Schmittgen, 2001) using glyceraldehyde-3-phosphate dehydrogenase (*Gapdh*) as the reference gene.

**Table 1:** Sequences of the primers used for quantitative reverse transcription-polymerase chain reaction

Gene	Forward	Reverse	NCBI Reference	Efficiency (%)	Amplicon size
*Psma3*	5'-AGA CCT TTT GGC TGC AGT TTC A-3'	5'-GCT TGC CTG GCT TTG CCA AT-3'	NM_017280.2	92.2	92
*Mrps14*	5'-TGC GTG ATG TGA AGA GAC GAA-3'	5'-TCA CCA GCC ATT TCC TGA AGG T-3'	NM_001105963.3	110.34	85
*Rps25*	5'-GCC GGA AAG TCG GCC AAA AA-3'	5'-TGC CTT TGG ACC ACT TCT TCT T-3'	NM_001005528.1	110.96	82
*Sec62*	5'-ACA AGA AGC GGA TCC AGG AAG T-3'	5'-AGA GGC ACT CCA CTG CTT TTG A-3'	NM_001393776.1	90.26	150
*Tnfɑ*	5'-GAC CCT CAC ACT CAG ATC ATC TTC T-3'	5'-TGC TTG GTG GTT TGC TAC GA-3'	NM_012675.3	91.77	70
*Tnfr1*	5'-TCT CCT CTA CGG ATC CCT CAA C-3'	5'-GCG ACG ACG TCT TCC CAT T-3'	XM_006237334.2	110.11	69
*Grp78*	5'-CGT CCA ACC CGG AGA ACA-3'	5'-ATT CCA AGT GCG TCC GAT G-3'	NM_013083.2	104.45	59
*Grp94*	5'-TCC TGC TGA CCT TCG GGT TT-3'	5'- AGG TCC TCT TCT ACC GTG CC-3'	NM_001012197.2	111.48	70
*Gapdh*	5'-GGG CAG CCC AGA ACA TCA-3'	5'-TGA CCT TGC CAC AGC CT-3'	NM_017008	98.67	91

### Bioinformatic analysis

Raw reads (fastq format) were processed using in-house Perl scripts, and paired-end clean reads were aligned to the reference genome using Hisat2 v2.05 (Mortazavi et al., 2008). The mapped reads of each sample were assembled by StringTie (v1.3.3b) (Pertea et al., 2015) in a reference-based approach, and FeatureCounts v1.5.0-p3 (Liao et al., 2014) was used to count the number of reads mapped to each gene. Differential expression analysis was performed using the DESeq2R package (1.20.0) (Anders and Huber, 2010). Gene Ontology (GO) enrichment analysis of the differentially expressed genes was carried out using the clusterProfiler package in R v4.4.0, correcting for gene length bias (Young et al., 2010). Only GO terms with a corrected *P* < 0.05 were considered to be significantly enriched.

### Western blotting

Protein extracted from the S1FL ischemic brain hemisphere was resuspended in 8 M urea with 4% sodium dodecyl sulfate (SDS) and a complete protease inhibitor cocktail (EDTA free) and were quantified using the BCA protein assay kit (Thermo Fisher Scientific, Cat# 23225). Electrophoresis was performed in 4%–12% bis-tris plus pre-cast gels (Invitrogen, Waltham, MA, USA, Cat# NW04127BOX) using 15 μg of each sample in a mini gel tank (Invitrogen, Cat# A25977). The separated protein was transferred to a nitrocellulose membrane (Thermo Fisher Scientific, Cat# 88014) by a semi-dry transfer system (Hoefer, Bridgewater, MA, USA, Cat# TE 77 PWR). The membrane was incubated with the Intercept Protein-free Blocking Solution (LI-COR, Lincoln, NE, USA, Cat# 927-80001) to block nonspecific protein binding. Then, the membrane was incubated overnight at 4°C, with the appropriate primary antibody diluted in Intercept: rabbit anti-phospho-eIF2α (1:1500, Abcam, Cambridge, UK, Cat# ab32157, RRID: AB_732117), rabbit anti-polyubiquitin (1:1000, Dako, Santa Clara, CA, USA, Cat# Z0458, RRID: AB_2315524), and mouse anti-GAPDH (1:1000, Abcam, Cat# ab8245, RRID: AB_2107448). After incubation, the membrane was washed with 20 mM Tris (Sigma-Aldrich, Cat# 252859), 150 mM NaCl, 0,1% Tween 20, and then incubated at room temperature for 75 minutes in the appropriate fluorescent secondary antibody: goat anti-rabbit IRDye 800 CW (1:20,000, LI-COR, Cat# 926-32211, RRID: AB_621843) and goat anti-mouse IRDye 680 RD (1:20,000, LI-COR, Cat# 2926-68070, RRID: AB_2651128) diluted in Intercept. The membrane was also stained with Revert 700 (LI-COR, Cat# 926-11015) following the manufacturer’s instructions for use as a loading control (Pillai-Kastoori et al., 2020). Briefly, images were acquired at 700 nm using a LICOR fluorescence scanner and quantified with Image Studio software (LICOR). For each sample, the signal intensity of the protein of interest was normalized to the total protein signal of the corresponding lane (Revert 700), thereby correcting for lane-to-lane variability in loading and transfer, and ensuring accurate, reproducible quantification of relative protein expression.

### Statistical analysis

No statistical methods were used to predetermine sample sizes; however, our sample sizes are similar to those reported in previous publications (Ugidos et al., 2023). GraphPad Prism 6 (GraphPad Software, Boston, MA, USA, www.graphpad.com) was used to perform the statistical analysis. The infarct volume was analyzed using two-way analysis of variance followed by Tukey’s *post hoc* test. The behavioral test results were analyzed with the Mann-Whitney *U* test or an unpaired *t*-test with Welch’s correction. The qRT-PCR and western blotting results were analyzed with one-way analysis of variance followed by Tukey’s *post hoc* test. In addition, Student’s *t*-test was used to compare the qRT-PCR and western blotting results between the sham experimental group and the mild and moderate ischemia experimental group. For all tests, a *P*-value of < 0.05 was considered statistically significant. For transcriptomics, statistical analysis was performed to identify significant differences between the different conditions. In the first step, the raw read count was normalized to correct the sequencing depth. Then, a statistical model was used to calculate the *P*-value. Finally, multiple hypothesis test corrections were used to obtain the false-discovery rate value (Anders and Huber, 2010).

## Results

### The infarct volume does not mirror the ischemia time

**Figure 2:**
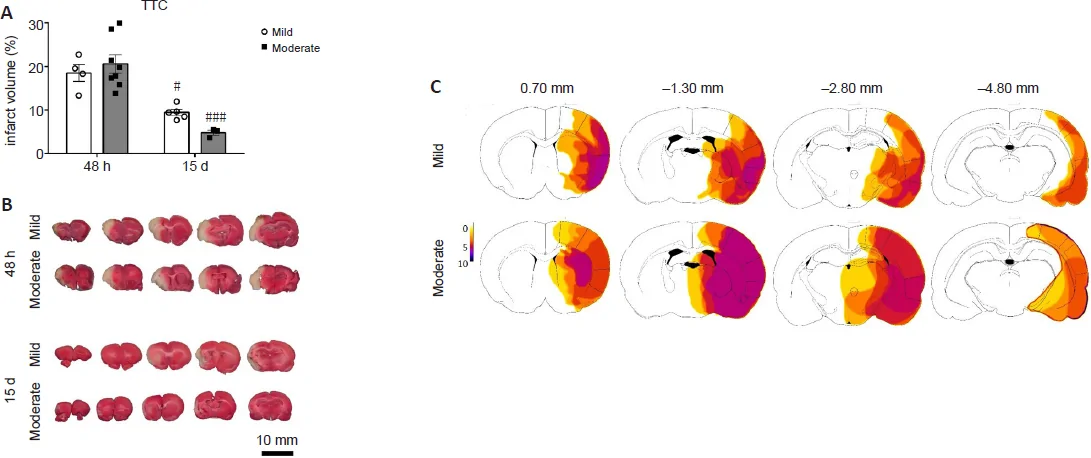
The infarct volume and lesion distribution of rats. (A) The percentage of infarct volume for mild and moderate ischemia after 48 hours and 15 days of reperfusion. #*P* < 0.05, ###*P* < 0.001, *vs*. 48 hours (Two-way analysis of variance followed by Tukey’s *post hoc* test). (B) Representative images of brain slices stained with 2,3,5-triphenyl tetrazolium chloride (TTC). White (or unstained) areas represent the infarcted regions. (C) A heatmap of the lesion distribution after 48 hours of reperfusion (*n* = 6). In the heatmap, yellow corresponds to areas where only one rat showed the infarct area detected by TTC, whereas dark purple indicates areas where all six rats showed the infarct area. Coordinates are related to bregma.

To assess the temporal evolution of tissue damage following mild and moderate MCAO, infarct volumes were compared at 48 hours and 15 days of reperfusion. The infarct volume was similar after mild and moderate ischemia when compared after 48 hours or 15 days of reperfusion (**Figure 2A**). After 48 hours of reperfusion, TTC staining showed a strong malfunction of the mitochondrial respiratory chain. After 15 days of reperfusion, the infarct volume for both mild (*P* = 0.0301) and moderate ischemia (*P* = 0.0003) had decreased significantly compared with that observed after 48 hours of reperfusion (**Figure 2A** and **B**). After 15 days of reperfusion, moderate ischemia produced a decrease in the infarct volume compared with mild ischemia, although the results were not significant (**Figure 2A** and **C**).

Together, these data demonstrate that, although both occlusion times yield comparable infarct sizes acutely, substantial spontaneous lesion decrease occurs over 15 days, with a nonsignificant trend toward greater recovery after moderate ischemia.

### Behavioral assays revealed greater cognitive and behavioral impairment associated with a longer ischemia time

To evaluate differences in the neurological deficit between the mild and moderate ischemia groups, we performed a battery of well-established behavioral tests to measure motor and/or sensory functions at different time points (2, 4, 7, and 15 days) after ischemia (Balkaya et al., 2013b, 2018).

### Modified neurological severity scores after 2 days of reperfusion

**Figure 3:**
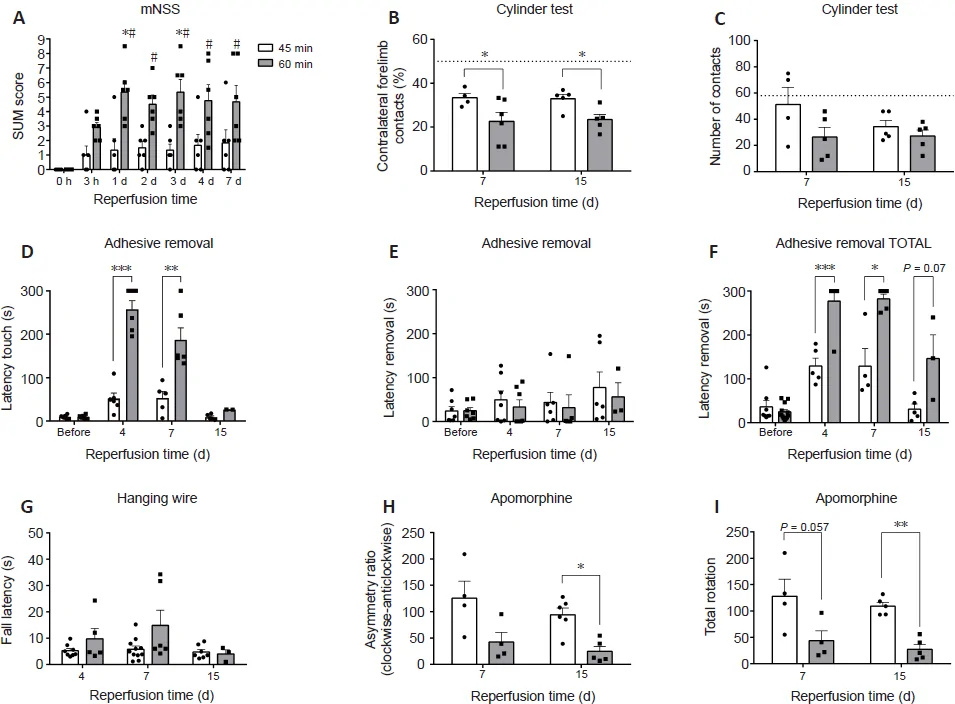
Behavioral test results of rats. (A) The modified neurological score (mNSS) was used to evaluate the neurological deficit, with a maximum score of 12. (B) Cylinder test. The percentage of contacts on the cylinder with the paw contralateral to the lesion measured after 7 and 15 days of reperfusion in mild ischemia (white bars) and moderate ischemia (gray bars). The dotted line represents the average total number of contacts on the cylinder with the forelimb contralateral to the lesion (left paw) in the sham group. (C) Cylinder test. The total number of contacts on the cylinder with the forelimb contralateral to the lesion (left paw) measured after 7 and 15 days or reperfusion in the mild and moderate ischemia groups. The dotted line represents the mean total number of contacts of the sham animals (both paws). (D) Adhesive removal test. The time (s) it took the mild and moderate ischemia rats to realize that it had adhesive tape attached to the contralateral paw before the intervention, after 4, 7, and 15 days of reperfusion. (E) Adhesive removal test. Time (s) it took the mild and moderate ischemia rats to remove the adhesive tape from the paw. (F) Adhesive removal test. The time (s) it took the mild and moderate ischemia rats to remove the sticker from its attachment. (G) Hanging wire test. The time (s) the mild and moderate ischemia rats could hang from a wire after 4, 7, and 15 days of reperfusion. (H) Apomorphine-induced rotation test. Motor asymmetry (expressed by subtracting the counterclockwise turns from the clockwise turns) of the mild and moderate ischemia groups after 7 and 15 days of reperfusion. (I) Apomorphine-induced rotation test. The total number of rotations the mild and moderate ischemia animals completed after 7 and 15 days of reperfusion. The data are presented as the mean ± standard error of the mean. *P < 0.05, **P < 0.01, ***P < 0.001; #*P* < 0.05, *vs.* 0 hours (Mann-Whitney *U* test or an unpaired *t*-test with Welch’s correction). All experiments were repeated ≥ 5 times.

After 2 days of reperfusion, rats with moderate ischemia displayed an mNSS > 4, while sham and mild ischemia rats presented an mNSS of 0–1 (**Figure 3A**).

### Cylinder test results

There was a significant decrease in the percentage of contralateral forelimb contacts with the cylinder after 7 and 15 days of reperfusion in the moderate ischemia group compared with the mild ischemia group (*P* = 0.047 at 7 days; *P* = 0.018 at 15 days), but there were no significant differences when we compared the outcome of mild and moderate ischemia rats over time (**Figure 3B**). There were no significant differences in the number of total contacts between the mild and moderate ischemia groups. Nonetheless, the mild ischemia group presented a slightly higher number of contacts than the moderate ischemia at both time points (**Figure 3C**).

### Adhesive removal test results

In the mild and moderate ischemia, the sensory deficit increased significantly after 4 and 7 days of reperfusion compared with the pre-ischemia values. However, this effect diminished gradually as the reperfusion time increased. The sensory deficit was significantly greater in the moderate ischemia group compared with the mild ischemia at all time points (**Figure 3D**). There was a similar response pattern for the total removal latency, with a significant increase after 4 and 7 days of reperfusion in the mild and moderate ischemia rats. The total latency of removal was significantly higher in the moderate ischemia compared with the mild ischemia from the fourth day after reperfusion (*P* = 0.007 at 4 days and *P* = 0.03 at 7 days; **Figure 3F**). When considering sensory and motor components independently, only the sensory component showed a significant difference between the mild and moderate ischemia group over time (*P* = 0.002 at 4 days and *P* = 0.004 at 7 days; **Figure 3D**). The motor component showed values similar to what were observed before MCAO in both the mild and moderate ischemia groups (**Figure 3E**).

### Wire hanging test results

There were no significant differences between the mild and moderate ischemia models (**Figure 3G**). Nevertheless, after 4 and 7 days of reperfusion, the moderate ischemia model tended to exhibit a longer hanging time compared with the mild ischemia model.

### Apomorphine-induced rotation test results

The rotational response to apomorphine was significantly higher in the mild ischemia group compared with the moderate ischemia group (*P* = 0.008). However, there were no significant changes in the total number of rotations during reperfusion (**Figure 3H**). There was a similar pattern in the asymmetry ratio (right turns – left turns). After 15 days of reperfusion, the moderate ischemia animals presented a significant decrease in the asymmetry ratio compared with the mild ischemia animals (*P* = 0.01; **Figure 3I**).

Overall, these functional assessments indicate that moderate ischemia produces more pronounced and sustained sensory–motor deficits, particularly in somatosensory processing, compared with mild ischemia, despite similar outcomes in motor strength and spontaneous limb use over the reperfusion period.

### Association of the ischemia time with gene expression changes

**Figure 4:**
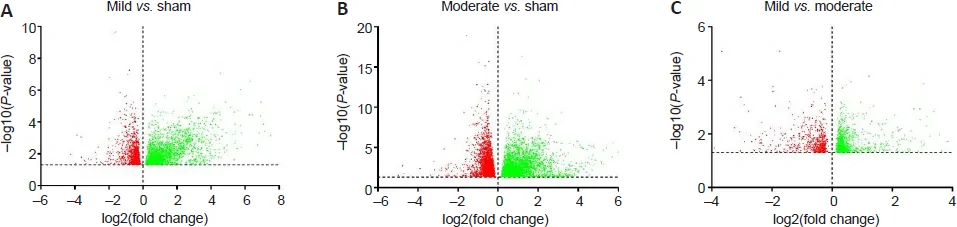
Differentially expressed genes (DEGs) between the conditions. (A–C) The volcano plots show the DEGs for the mild ischemia *versus* sham (A), moderate ischemia *versus* sham (B), and mild *versus* moderate ischemia (C). Downregulated genes are indicated in red, and upregulated genes are indicated in green. The threshold used to define a DEG was *P* < 0.05, *n* = 5 for each condition.

To elucidate the molecular consequences of a 15-minute shift in occlusion time, comprehensive transcriptomic profiling of brain tissue was performed following mild and moderate MCAO. Transcriptomic analysis revealed significant differences in the number of up- and downregulated genes between the mild and moderate ischemia models. The mild ischemia presented 2206 upregulated genes, and 1230 downregulated genes compared with the sham animals (**Figure 4A**), while the moderate ischemia group presented 3402 upregulated genes, and 2206 downregulated genes compared with the sham group (**Figure 4B**). The comparison between the mild and moderate ischemia groups revealed 895 upregulated and 623 downregulated genes in the mild ischemia rats compared with the moderate ischemia rats (**Figure 4C**).

**Figure 5:**
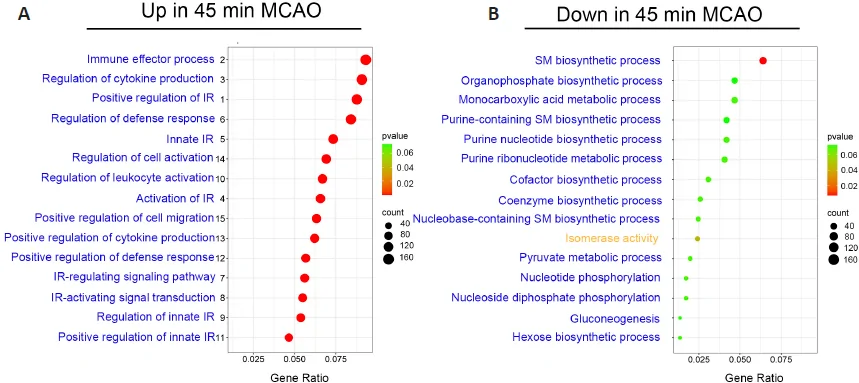
Gene Ontology (GO) enrichment analysis for upregulated (A) and downregulated pathways (B) in the cerebral ipsilateral cortex after 15 days of reperfusion in mild ischemia rats compared with sham rats. The y-axis shows the 15 most significant GO annotations (blue for biological processes and orange for molecular functions). The x-axis indicates the ratio of the number of differentially expressed genes linked with the GO term to the total number of differentially expressed genes. The point size represents the number of genes annotated to a specific GO term, and the point color indicates the adjusted *P*-value. IR: Immune response; MCAO: middle cerebral artery occlusion; SM: small molecule.

GO analysis comparing the mild ischemia and the sham groups revealed substantial transcriptomic signatures associated with mild ischemia. The 15 top upregulated GO terms in mild ischemia are related to biological processes, mainly the immune response (**Figure 5A** and **Additional Table 1**). The downregulated GO terms are related to the metabolism of small molecules, isomerase activity, and other metabolic processes (**Figure 5B** and **Additional Table 2**).

**Additional Table 1:** Gene Ontology (GO) enrichment analysis for upregulated pathways in the cerebral ipsilateral cortex after 15 days of reperfusion in mild ischemia rats compared with sham rats

Cat.	GOID	Descrip.	Gene Ratio	Bg ratio	*P* value	*P*_adj_	geneName	#
BP	GO:000 2252	Immune effector process	156/1675	476/ 14671	1.08E-22	2.69E-20	Cd180/C3/Tlr3/Itgam/Cd84/Dtx3l/Nlrp3/Itgal/Tlr8/Tnfsf4/Irf8/Cx3cr1/Lbp/Ptprc/Irgm/Adora3/Sting1/Gpr183/Mlkl/Rsad2/ RT1-Da/Dock2/Nckap1l/Irf7/Myo1g/Bcl3/Sash3/Slfn2/Ptpn6/Havcr2/Map3k7/Parp9/Rac2/Cgas/Unc93b1/C4a/Gbp2/ Fgr/Btk/Eif2ak2/Enpp3/Irf5/Tlr9/Vav1/Ctsc/Apbb1ip/Ticam2/Was/Hlx/Oas3/N18/Aim2/Cfb/Itch/Gbp7/Ifih1/Tre m2/ Rig1/Dock11/Slc11a1/Gpam/Serping1/Tlr2/Entpd7/Stat3/Spi1/Tlr7/N18r1/Fgl2/Dennd1b/Nlrc5/Lyn/Casp4/ G rn/Fes/Axl/Pycard/Trim56/Bst2/Inpp5d/Serpinb9/Syk/Dhx58/Anxa3/Cxd9/Lag3/Lacc1/C1qa/Rc3h1/Cd2/C1qc/ Fcnb/Samhd1/A2m/C6/Ifngr2/Ifitm2/N7r/Nmi/Dhx15/Lcp1/Abcc9/Pik3r6/Casp1/N1b/Tcirg1/Tnfrsf1b/Irf2/C4b/Itg ax/Atad5/ RT1-DMb/Kmt2a/Zc3h12a/Tlr4/Nfkbiz/Il18rap/F2rl1/B2m/Ifi35/Dock10/Zmpste24/Hfe/Appl2/Plcg2/Myd88/Unc13d/ Scimp/Mfng/Kdm5d/Klrk1/Il1r1/Rab27a/Nbn/Phf14/Ada/Usp15/Psen1/Cd81/Pla2g5/Clu/Pum1/Zbtb1/Irf1/Mmp12/ Abl1/Wnt5a/Jag1/Irak4/Ap1g1/Foxf1/Tp53/Stxbp2/Parp3/Xcl1/Ccr6	156
BP	GO:000 1817	Regulation of cytokine productio n	152/1675	491/ 14671	1.47E-18	2.44E-15	Clec7a/C3/Tlr3/N1rl1/Runx1/Oas2/C3ar1/Cd84/Nlrp3/Tlr8/Cd3/Serpinb1a/Tnfsf4/Card11/Irf8/Adcy7/Cx3cr1/ Gpr18/Lbp/Ptprc/F2r/Irgm/Sting1/Thbs1/Itgav/Rsad2/Cd74/Nckap1l/Irf7/Erbin/Bcl3/Sash3/Igf1/Rab7b/Nlrc4/ Gpnmb/Ptpn6/Havcr2/Ma p3k7/Csf1r/Cgas/Unc93b1/Fgr/Pld4/Btk/Itgb8/Cd14/Irf5/Tlr9/Ticam2/Oas3/N18/Aim2/ Tgf b1/Elf4/Ifih1/Trem2/Rig1/Nfam1/S1pr3/Slc11a1/Cmklr1/Gpam/Tlr2/Cyba/Stat3/Smad4/Tlr7/Casp8/Il18r1/ Denn d1b/Lyn/Casp4/Tsku/Axl/Cyp1b1/Pycard/Traf3ip3/Inpp5d/Syk/Elf1/Cd6/Dhx58/Bmpr1a/Tlr6/Lag3/Lacc1/Creb1/ Pml/Tyrobp/Cd2/Fcnb/Gbp5/Hspb1/Dhx9/Hif1a/Rbm47/Nmi/Casp1/N1b/Tnfrsf1b/Tlr1/Zc3h12a/Tlr4/Mmp8/F2rl 1/ Clec5a/Epha2/B2m/Lu m/Tmf1/Zfp36/Mertk/Adam17/Sulf1/Tmed10/Hfe/Rel/Appl2/Kpna6/Xiap/Plcg2/Myd88/Scimp/Nav3/Pik3r 1/Plin3/Klrk1/M1r1/Cd274/Tnfrsf21/Pde4d/Usp15/Psen1/Cd81/Foxp1/Clu/Ppara/Yy1/Gsdm d/ Pum1/Irf1/Adra2a/Mmp12/Btn2a2/Abl1/Wnt5a/Rac1/Lgr4/Cd34/Hpse/Xcl1	152
BP	GO:005 0778	Positive regulation of immune response	147/1675	421/ 14671	3.58E-24	1.78E-20	C3/Tlr3/Nfatc2/C3ar1/Itgam/Nlrp3/Tlr8/Tnfsf4/Card11/Lbp/Ptprc/Irgm/Adora3/Sting1/Tlr13/Rsad2/ RT1-Da/Nckap1l/Irf7/Erbin/Zbp1/Myo1g/Sash3/Rab7b/Klhl6/Trim25/Ptpn6/Havcr2/Map3k7/Tasl/Parp9/LOC1025560 96/Cgas/Unc93b1/C4a/Gbp2/Fgr/Otud4/Slc15a3/Wdfy1/Btk/Cd14/Kcnn4/Themis2/Irf5/Tlr9/Vav1/Lcp2/Ticam2/ Tnfsf13b/Hlx/Oas3/N18/Aim2/Cfb/Alpk1/Sh2b2/Pik3ap1/Gbp7/Cd22/Ifih1/Trem2/Gbp4/Rig1/Nfam1/Cd38/ Slc11a1/Serping1/Tlr2/Cyba/Blnk/Spi1/Tlr7/Rps6ka3/N18r1/Dennd1b/Nlrc5/Lyn/Casp4/Cmtm3/Pycard/Trim56/Syk/ Elf1/Dhx58/Tlr6/Lag3/Lacc1/C1qa/Rc3h1/Tyrobp/C1qc/Fcnb/Gbp5/Cd8a/Mapkapk3/Cblb/A2m/C6/Rbm47/Nmi/ Tlr12/Casp1/Naglu/ENSRNOG00000069878/Tlr1/C4b/Atad5/RT1-DMb/ Fyn/Zc3h12a/Tlr4/Nfkbiz/N18rap/F2rl1/B2m/Ifi35/Cacnb4/Appl2/Xiap/Blk/Plcg2/Myd88/Scimp/Plin3/Klrk1/N1r1/Cd274/Mnda/Tnfrsf21 /Ada/Pla2g4a/Pde4d/Usp15/Psen1/Cd81/Foxp1/Pum1/Zbtb1/Mmp12/Btn2a2/Abl1/Wnt5a/Lpxn/Ap1g1/Lgr4/Xcl1	147
BP	GO:003 1347	Regulation ofdefense response	141/1675	485/ 14671	4.11E-13	3.40E-10	Clec7a/C3/Tlr3/N1rl1/Dtx3l/Nlrp3/Tlr8/Cd3/Serpinb1a/Tnfsf4/Ythdf3/Adcy7/Lbp/Irgm/Adora3/Sting1/Tlr13/Rsa d2/Socs3/ Irf7/Erbin/Zbp1/Rab7b/Trim25/Ptpn6/Havcr2/Tasl/Parp9/LOC102556096/Cgas/Tnfrsf1a/Unc93b1/Gb p2/ Fgr/Otud4/Slc15a3/Wdfy1/Pld4/Trim21/Cd14/Enpp3/Tlr9/Vav1/Ticam2/Oas3/N18/Aim2/Alpk1/Elf4/Pik3ap1/Itch/Gbp7/Ifih1/Trem2 /Gbp4/Rig1/Ets1/Serping1/Parp14/Tlr2/Cyba/Aoah/Stat3/Spi1/Tlr7/Rps6ka3/Fgl2/Nlrc5/ Lyn/Casp4/Abi3bp/Grn/Pycard/Trim56/Syk/Cd6/Dhx58/Tlr6/Lag3/Lacc1/Tyrobp/Fcnb/Gbp5/Mapkapk3/Akna/ Hif1a/Samhd1/A2m/Adamts12/Rbm47/Nmi/Gpr17/Tlr12/N10ra/Pik3r6/Casp1/Tnfaip8l2/N1b/Tnfaip6/Nfe2l2/ Tnfrsf1b/Naglu/ENSRNOG00000069878/Cdh5/Tlr1/Zc3h12a/Tlr4/Nfkbiz/Il18rap/F2rl1/Ifi35/Zfp36/Zmpste24/Appl2/ Kpna 6/Rictor/Xiap/Plcg2/Myd88/Scimp/Pbk/Cd47/Plin3/Klrk1/Il1r1/Mnda/Ada/Fanca/Usp15/Foxp1/Ppara/Gsdmd/Pum1/Ppp6c/ Gpsm3/Rb1/Mmp12/Wnt5a/Ap1g1/Foxf1/Lgr4	141
BP	GO:004 5087	Innate immune response	123/1675	372/ 14671	1.95E-16	1.94E-12	Tlr3/Tlr8/Serpinb1a/Irf8/Ythdf3/Cx3cr1/Lbp/Irgm/Sting1/Tlr13/Rsad2/Cd74/Irf7/Erbin/Zbp1/Uba7/Rab7b/Trim25/Ptpn6/ Havcr2/Cxcl16/Tasl/Parp9/LOC102556096/Cgas/Unc93b1/Gbp2/Fgr/Otud4/Slc15a3/Wdfy1/Nlrp1a/ Tri m21/Cd14/Irf5/Tlr9/Vav1/Ch25h/Ticam2/Was/RT1-Ba/Oas3/Il18/Aim2/Alpk1/Pik3ap1/Gbp7/Ifih1/Trem2/ Gbp4/Rig1/Trim26/Slc11a1/Serping1/Parp14/Tlr2/Cyba/Spi 1/Tlr7/Rps6ka3/Nlrc5/Lyn/Grn/Pycard/Trim56/Syk/Dhx58/Tlr6/Lag3/ Lacc1/Aqp4/Pml/Tyrobp/Cd2/Fcnb/Gbp5/Mapkapk3/Samhd1/Ifngr1/Rab43/Cybb/A2m/Eprs1/Ifngr2/Rbm47/Nmi/Tlr12/Pik3r6/ Ube2l6/Slc22a5/Nfe2l2/N aglu/ENSRNOG00000069878/Tlr1/Tlr4/Nfkbiz/N18rap/F2rl1/aec5a/Ifi35/Gch1/Ciita/Appl2/Xiap/Plcg2/ Myd88/Vi m/Unc13d/Scimp/Plin3/Klrk1/Rab27a/Mnda/Usp15/Mid2/Pum1/Ppp6c/Mmp12/Wnt5a/Ap1g1/Tp53/Lgr4/Pde1 2	123
BP	*GO:005* 0865	Regulation of cell activation	116/1675	395/ 14671	7.65E-09	2.72E-06	Tafa 3/Clec7a/Il1rl1/Nfatc2/Itgam/Cd84/Igfbp2/Nlrp3/Tnfsf4/Card11/Lbp/Sox4/Ptprc/Irgm/Adora3/Thbs1/Gpr18 3/Dock8/Cd74/RT1- Da/Nckap1l/Sash3/Igf1/Ptpn6/Havcr2/Rac2/Cgas/Fgr/Rasal3/Shh/Zfp36l2/Lgals3/Btk/Enpp3/Themis2/Tlr9/Ctsc/ Tnfsf13b/Itpkb/RT1- Ba/Hlx/Il18/Mmp14/Tgfb1/Itch/Trem2/Nfam1/Cd38/Rhoh/Gpam/Tgfbr2/Sfrp1/Spi1/Fgl2/Lyn/Prdm1/Gm/Fes/A xl/Pycard/Samsn1/Inpp5d/Syk/Plek/RT1- DOa/Cd6/Tnfrsf13b/Atm/Lag3/Rc3h1/Tyrobp/Ccl2/Cblb/M15ra/N7r/Zfp36l1/Vcam1/Pag1/Pik3r6/Tnfaip8l2/ENSR NOG00000071219/Tnfrsf1b/Prkca/Atad5/RT1- DMb/Zc3h12a/Tlr4/Nfkbiz/Mmp8/F2rl 1/Pde5a/Alox12/Mertk/Hfe/Casp3/Myd88/Unc13d/Il6st/Sh2b3/Atp11c/C d274/Zeb1/Tnfrsf21/Ada/Pla2g4a/Fanca/Cd81/Zbtb1/Btn2a2/Abl1/Wnt5a/Ap1g1/Foxf1/Sos2/Parp 3/Xcl1	116
BP	GO:000 2694	Regulation of leukocyte activation	112/1675	365/ 14671	8.26E-10	4.10E-07	Tafa 3/Clec7a/Il1rl1/Nfatc2/Itgam/Cd84/Igfbp2/Nlrp3/Tnfsf4/Card11/Lbp/Sox4/Ptprc/Irgm/Adora3/Thbs1/Gpr18 3/Dock8/Cd74/RT1-Da/Nckap1l/Sash3/Igf1/Ptpn6/Havcr2/Rac2/Cgas/Fgr/Rasal3/Shh/Zfp36l2/Lgals3/Btk/Enpp3/Themis2/Tlr9/ Ctsc/Tnfsf13b/Itpkb/RT1-Ba/Hlx/Il18/Mmp14/Tgfb1/Itch/Trem2/Nfam1/Cd38/Rhoh/Gpam/Tgfbr2/Sfrp1/Spi1/Fgl2/ Lyn/Prdm1/Gm/Fes/Axl/Pycard/Samsn1/Inpp5d/Syk/RT1-DOa/Cd6/Tnfrsf13b/Atm/Lag3/Rc3h1/Tyrobp/Cd2/Cblb/N15ra/ N7r/Zfp36l1/Vcam1/Pag1/Pik3r6/Tnfaip8l2/ENSRNOG00000071219/Tnfrsf1b/Atad5/RT1-DMb/Zc3h12a/Tlr4/Nfkbiz/Mmp8/ F2rl1/Pde5a/Mertk/Hfe/Casp3/Myd88/Unc13d/Il6st/Atp11c/Cd274/Zeb1/Tnfrsf21/Ada/Pla2g4a/Fanca/Cd81/Zbtb1/Btn2a2/Abl1/ Wnt5a/Ap1g1/Foxf1/Sos2/Parp3/Xcl1	112
BP	GO:000 2253	Activation of immune response	110/1675	292/ 14671	4.54E-19	5.64E-15	C3/Tlr3/Nfatc2/C3ar1/Tlr8/Card11/Lbp/Ptprc/Sting1/Tlr13/Rsad2/Nckap1l/Irf7/Erbin/Myo1g/Rab7b/Klhl6/Trim25/Ptpn6/ Havcr2/Tasl/LOC102556096/Cgas/Unc93b1/C4a/Gbp2/Otud4/Slc15a3/Wdfy1/Btk/Cd14/Kcnn4/Themis2/Tlr9/Lcp2/Ticam2/Oas3/ Aim2/Cfb/Alpk1/Sh2b2/Pik3ap1/Gbp7/Cd22/Ifih1/Trem2/Gbp4/Rig1/Nfam1/Cd38/Serping1/Tlr2/Cyba/Blnk/Tlr7/Rps6ka3/Dennd1b/ Lyn/Cmtm3/Pycard/Syk/Elf1/Dhx58/Tlr6/Lacc1/C1qa/Rc3h1/Tyrobp/C1qc/Fcnb/Gbp5/Cd8a/Mapkapk3/Cblb/A2m/C6/Nmi/Tlr12/ Naglu/ENSRNOG00000069878/Tlr1/C4b/Fyn/Zc3h12a/Tlr4/Nfkbiz/F2rl1/Ifi35/Cacnb4/Appl2/Xiap/Blk/Plcg2/Myd88/Scimp/Plin3/ Klrk1/Mnda/Tnfrsf21/Ada/Pde4d/Usp15/Psen1/Cd81/Foxp1/Pum1/Btn2a2/Abl1/Lpxn/Lgr4	110
BP	GO:003 0335	Positive regulation of cell migration	106/1675	349/ 14671	3.35E-08	1.11E-05	Clec7a/Plau/C3ar1/Col1a1/Tgfbr1/Cx3cr1/Lbp/Ptprc/F2r/Adora3/Thbs1/Itgav/Dock8/Nckap1l/Igf1/Cxcl10/Pik3cg /Cxcl 16/Csf1r/Rac2/Fgr/Foxc2/Lgals3/Cd40/Mmp14/Tgfb1/Notch1/Trem2/Prox1/Itga6/Glipr2/Ets1/Cmklr1/Ano 6/ Tgfbr2/Stat3/Hmox1/Spi1/Lyn/Syde1/Gm/Atm/Angpt4/Anxa3/Stk4/Ccl2/Jam2/Arhgap5/Hspb1/Adam9/Hif1a/ Atp8a 1/Aqp1/Cldn1/Ctsh/Pdgfrb/Nipbl/Gcnt2/Met/Adgra2/Vtn/Cdh5/Prkca/Itgax/Zc3h12a/Adam10/Dab2/Tlr4/F2rl1/ Epha2/Fut4/Bmpr2/Hbegf/Gcnt1/Lgmn/Mospd2/Adam17/Sema6d/Plaa/Cass4/Rin2/Lrrc15/Spag9/Numb/Pik3r1/Bmp7/Sash1/Il1r1/ Mgat5/Pde4d/Foxp1/Adra2a/Sparc/Pik3c2a/Abl1/Adamts1/Wnt5a/Apc/Cpne3/Rac1/Zfp609/Foxf1/Nrp1/Epb41l4b/Xcl1/Sp1	106
BP	GO:000 1819	Positive regulation ofcytokine productio n	104/1675	329/ 14671	2.72E-09	1.04E-06	Clec7a/C3/Tlr3/N1rl1/Runx1/Oas2/C3ar1/Nlrp3/Tlr8/Ccl3/Tnfsf4/Card11/Irf8/Lbp/F2r/Sting1/Thbs1/Rsad2/Cd74 /I rf7/Bcl 3/Sash3/Rab7b/Nlrc4/Havcr2/Map3k7/Csf1r/Cgas/Unc93b1/Fgr/Cd14/Irf5/Tlr9/Ticam2/Oas3/N18/Aim2 /Tgfb1/Ifih1/Trem2/Rig1/Nfam1/Slc11a1/Tlr2/Cyba/Stat3/Tlr7/Casp8/Il18r1/Dennd1b/Casp4/Cyp1b1/Pycard/Traf3ip3/ Syk/Cd6/Dhx58/Bmpr1a/Lacc1/Creb1/Fcnb/Gbp5/Hspb1/Dhx9/Hif1a/Rbm47/Casp1/Il1b/Tlr1/Tlr4/Mmp8/F2rl1/Clec5a/B2m/Lum/ Tmf1/Adam17/Sulf1/Tmed10/Kpna6/Xiap/Plcg2/Myd88/Scimp/Pik3r1/Plin3/Klrk1/Il1r1/Cd274/Pde4d/Usp15/Psen1/Cd81/Clu/ Gsdmd/Pum1/Irf1/Adra2a/Mmp12/Abl1/Wnt5a/Cd34/Hpse/Xcl1	104
BP	GO:003 1349	Positive regulation of defense response	95/1675	282/ 14671	1.29E-10	5.34E-07	Clec7a/C3/Tlr3/Il1rl1/Nlrp3/Tlr8/Cd3/Lbp/Irgm/Adora3/Sting1/Tlr13/Rsad2/Irf7/Erbin/Zbp1/Rab7b/Trim25/Hav cr2/Tasl/Parp9/LOC102556096/Cgas/Unc93b1/Gbp2/Otud4/Slc15a3/Wdfy1/Cd14/Tlr9/Vav1/Ticam2/Oas3/Il18/ Aim2/Alpk1/Pik3ap1/Gbp7/Ifih1/Trem2/Gbp4/Rig1/Ets1/Tlr2/Cyba/Stat3/Spi1/Tlr7/Rps6ka3/Nlrc5/Lyn/Casp4/P ycard/Trim56/Syk/Cd6/Dhx58/Tlr6/Lag3/Lacc1/Tyrobp/Fcnb/Gbp5/Mapkapk3/Hif1a/Rbm47/Nmi/Tlr12/Casp1/Il 1b/Naglu/ENSRNOG00000069878/Tlr1/Tlr4/Nfkbiz/Il18rap/F2rl1/Ifi35/Appl2/Kpna6/Xiap/Plcg2/Myd88/Scimp/C d47/Plin3/Klrk1/Mnda/Usp15/Gsdmd/Pum1/Mmp12/Wnt5a/Ap1g1/Lgr4	95
BP	GO:000 2764	Immune responseregulating s ignaling pa thway	94/1675	258/ 14671	2.91E-12	1.88E-09	Tlr3/Nfatc2/C3ar1/Tlr8/Card11/Lbp/Ptprc/Sting1/Tlr13/Rsad2/Nckap1l/Irf7/Erbin/Myo1g/Rab7b/Klhl6/Trim25/Ptpn6/Ha vcr2/Tasl/Lat2/Cgas/Unc93b1/Otud4/Slc15a3/Wdfy1/Btk/Cd14/Kcnn4/Themis2/Tlr9/Lcp2/Ticam2/Oas3 /Alpk1/Sh2b2/Pik3ap1/Cd22/Ifih1/Trem2/Rig1/Nfam1/Cd38/Tlr2/Cyba/Blnk/Tlr7/Rps6ka3/Dennd1b/Lyn/Cmtm3/Syk/Elf1/Dhx58/Tlr 6/Lacc1/Rc3h1/Tyrobp/Fcnb/Cd8a/Mapkapk3/Cblb/Nmi/Tlr12/Ctsh/Naglu/Tlr1/Fyn/Zc3h12a/Tlr4/Nfkbiz/F2rl1/Ifi35/Cacnb4/Appl2/ Xiap/Blk/Plcg2/Myd88/Scimp/Plin3/Klrk1/Tnfrsf21/Ada/Pde4d/Usp15/P sen1/Cd81/Foxp1/Pum1/Btn2a2/Abl1/Lpxn/Lgr4	94
BP	GO:000 2757	Immune responseactivating s ignal transducti on	92/1675	249/ 14671	3.02E-12	1.88E-09	Tlr3/Nfatc2/C3ar1/Tlr8/Card11/Lbp/Ptprc/Sting1/Tlr13/Rsad2/Nckap1l/Irf7/Erbin/Myo1g/Rab7b/Klhl6/Trim25/Ptpn6/ Ha vcr2/Tasl/Cgas/Unc93b1/Otud4/Slc15a3/Wdfy1/Btk/Cd14/Kcnn4/Themis2/Tlr9/Lcp2/Ticam2/Oas3/Alpk1/Sh2b2/ Pik3ap1/Cd22/Ifih1/Trem2/Rig1/Nfam1/Cd38/Tlr2/Cyba/Blnk/Tlr7/Rps6ka3/Dennd1b/Lyn/Cmtm3/Syk/ Elf1/Dhx58/Tlr6/Lacc1/Rc3h1/Tyrobp/Fcnb/Cd8a/Mapkapk3/Cblb/Nmi/Tlr12/Naglu/Tlr1/Fyn/Zc3h12a/Tlr4/Nfkbiz/ F2rl1/Ifi35/Cacnb4/Appl2/Xiap/Blk/Plcg2/Myd88/Scimp/Plin3/Klrk1/Tnfrsf21/Ada/Pde4d/Usp15/Psen1/Cd81/Foxp1/Pum1/Btn2a2/ Abl1/Lpxn/Lgr4	92
BP	GO:004 5088	Regulation of innate immune response	90/1675	245/ 14671	1.73E-11	9.55E-09	Tlr3/Tlr8/Serpinb1a/Ythdf3/Lbp/Irgm/Sting1/Tlr13/Rsad2/Irf7/Erbin/Zbp1/Rab7b/Trim25/Havcr2/Tasl/Parp9/ LOC102556096/Cgas/Unc93b1/Gbp2/Fgr/Otud4/Slc15a3/Wdfy1/Trim21/Cd14/Tlr9/Vav1/Ticam2/Oas3/Aim2/ Alpk 1/Pik3ap1/Gbp7/Ifih1/Trem2/Gbp4/Rig1/Serping1/Parp14/Tlr2/Cyba/Spi1/Tlr7/Rps6ka3/Nlrc5/Lyn/Grn/Pycard/ Trim56/Syk/Dhx58/Tlr6/Lag3/Lacc1/Tyrobp/Fcnb/Gbp5/Mapkapk3/Samhd1/A2m/Rbm47/Nmi/Tlr12/Pik3r6/Nfe2l2/ Naglu/ENSRNOG00000069878/Tlr1/Tlr4/Nfkbiz/Il18rap/F2rl1/Ifi35/Appl2/Xiap/Plcg2/Myd88/Scimp/Plin3/ Klrk1/Mnda/Usp15/Pum1/Ppp6c/Mmp12/Wnt5a/Ap1g1/Lgr4	90
BP	GO:004 5089	Positive regulation ofinnate immune response	78/1675	203/ 14671	1.11E-09	5.02E-07	Tlr3/Tlr8/Lbp/Irgm/Sting1/Tlr13/Rsad2/Irf7/Erbin/Zbp1/Rab7b/Trim25/Havcr2/Tasl/Parp9/LOC102556096/Cgas/ Unc93b1/Gbp2/Otud4/Slc15a3/Wdfy1/Cd14/Tlr9/Vav1/Ticam2/Oas3/Aim2/Alpk1/Pik3ap1/Gbp7/fih1/Trem2/ Gbp4/Rig1/Tlr2/Cyba/Spi1/Tlr7/Rps6ka3/Nlrc5/Lyn/Pycard/Trim56/Syk/Dhx58/Tlr6/Lag3/Lacc1/Tyrobp/Fcnb/Gbp5/Mapkapk3/ Rbm47/Nmi/Tlr12/Naglu/ENSRNOG00000069878/Tlr1/Tlr4/Nfkbiz/Il18rap/F2rl1/Ifi35/Appl2/Xiap/ Plcg2/Myd88/Scimp/Plin3/Klrk1/Mnda/Usp15/Pum1/Mmp12/Wnt5a/Ap1g1/Lgr4	78

Cat.: Category; Descrip.: description; #: counts. Note: Category: Classification of GO databases, including biological processes (BP), cellularcomponents(CC), molecularfunction(MF).GOID: Unique identification id of Gene Ontology database. Description: Function description correspondingtothe GO number. GeneRatio: Ratio between the numberof differentially expressed genes in each GO term and all differentially expressed genes that can be found in GO database. BgRatio: In background GO database, the ratio of all genes concerning this GO term to all genes. *P* value: Statistics category term; abbreviation for probability value. *P*_adj_: Adjusted p-value. Generally, GO Terms with Corrected_*P* value <0.05 are significant enrichment. geneName: Differentially expressed genes in this term. Count: The numberof difference gene annotated to GO number.

**Additional Table 2:** Gene Ontology (GO) enrichment analysis for downregulated pathways in the cerebral ipsilateral cortex after 15 days of reperfusion in mild ischemia rats compared with sham rats

Cat.	GOID	Descrip.	Gene ratio	Bg ratio	*P* value	*P*_adj_	geneName	#
BP	GO:004 4283	Small molecule biosynthetic process	52/813	473/ 14671	1,73E+08	0.0068441 33447987 88	Pmvk/Alox15/Nme5/Tm7sf2/Ass1/Gstm7/C1qtnf12/Pck2/Coq8b/Aloxe3/Eno1/Pde9a/Gck/Cftr/Dkk3/ ENSRNOG0 0000063843/Tecr/Bhmt/LOC500959/Csad/G6pc3/Nme1/Gpi/Itpa/Tpi1/Ip6k2/Uox/Snca/Scap/Sds/Nags/Spr/ Npd c1/Stk11/Pdk2/Bpgm/Itpkc/Dut/Prkag2/Acot7/Prkaca/Wdr5/Coq10a/Mcat/Acsf3/Actn3/Pklr/Coq6/Pgam1/Adsl/ Uros/Coq8a	52
BP	GO:009 0407	Organophosphate biosynthetic process	38/813	378/ 14671	0.0002787 84185344 252	0.0728533 94154686 9	Alox15/Pcyt2/Nme5/Ampd2/Ppcs/Eno1/Nppa/Gck/ENSRNOG00000063843/LOC500959/Cryl 1/Dhrs7b/Nme1/ Atp5me/Gpi/Tpi1/Mboat7/Mvd/Ip6k2/Snca/Ptdss2/AABR07051450.1/Bpgm/Itpkc/Dut/Prkag2/Adgrv1/Acot7/ Prkaca/Nppc/Pgs1/Actn3/Pklr/Pgam1/Fabp3/Ckmt1/Ormdl3/Adsl	38
BP	GO:003 2787	Monocarboxylic acid metabolic process	38/813	371/ 14671	0.0001912 53276826 507	0.0698459 77229872 5	Pex5/Alox15/Grhpr/Gstm7/Aloxe3/Eno1/Gck/Arv1/Aig1/ENSRNOG00000063843/Abhd16a/Tecr/LOC500959/Cryl 1/ Gpi/Tpi1/Scap/Sds/Alkbh7/Pdk2/Lypla2/Bpgm/Tysnd1/Slc27a4/Prkag2/Acot7/Prkaca/Fahd1/Ldhd/Mtln/Mcat /Gpx4/Acsf3/Me3/Actn3/Pklr/Pgam1/Fabp3	38
BP	GO:007 2521	Purine- containing compound metabolic process	34/813	323/ 14671	0.0002463 98244882 295	0.0728533 94154686 9	Nme5/Ampd2/Myh3/Ndufb8/Ppcs/Eno1/Nppa/Pde9a/Gck/ENSRNOG00000063843/LOC500959/Myh8/Sult2b1/ Nme1/Atp5me/Gpi/Tpi1/Uox/Ndufa8/AABR07051450.1/Bpgm/Prkag2/Guk1/Adgrv1/Acot7/Prkaca/Nppc/ Ndufv 1/Myh7/Ndufs7/Actn3/Pklr/Pgam1/Adsl	34
BP	GO:000 6164	Purine nucleotide biosynthetic process	22/813	180/ 14671	0.0004052 98205536 122	0.0795501 09067207	Nme5/Ampd2/Ppcs/Eno1/Nppa/Gck/ENSRNOG00000063843/LOC500959/Nme1/Atp5me/Gpi/Tpi1/Bpgm/Prkag2/ Adgrv1/Acot7/Prkaca/Nppc/Actn3/Pklr/Pgam1/Adsl	22
BP	GO:000 9152	Purine ribonucleotide biosynthetic process	22/813	179/ 14671	0.0003748 53008949 281	0.0795501 09067207	Nme5/Ampd2/Ppcs/Eno1/Nppa/Gck/ENSRNOG00000063843/LOC500959/Nme1/Atp5me/Gpi/Tpi1/Bpgm/Prkag 2/ Adgrv1/Acot7/Prkaca/Nppc/Actn3/Pklr/Pgam1/Adsl	22
BP	GO:005 1188	Cofactor biosynthetic process	25/813	195/ 14671	7.79E+09	0.0698459 77229872 5	Ppcs/Coq8b/Eno1/Fech/Gck/ENSRNOG00000063843/LOC500959/Gpi/Tpi1/Alad/Spr/Pdk2/Bpgm/Alas2/Prkag2/ Acot7/Prkaca/Coq10a/Actn3/Pklr/Coq6/Pgam1/Uros/Mat2a/Coq8a	25
BP	GO:000 9108	Coenzyme biosynthetic process	21/813	159/ 14671	0.0001871 40184090 13	0.0698459 77229872 5	Ppcs/Coq8b/Eno1/Gck/ENSRNOG00000063843/LOC500959/Gpi/Tpi1/Spr/Pdk2/Bpgm/Prkag2/Acot7/Prkaca/ Coq 10a/Actn3/Pklr/Coq6/Pgam1/Mat2a/Coq8a	21
BP	GO:003 4404	Nucleobase- containing small molecule biosynthetic process	20/813	149/ 14671	0.0002119 75651683 983	0.0698459 77229872 5	Nme5/Eno1/Pde9a/Gck/ENSRNOG00000063843/LOC500959/Nme1/Gpi/Itpa/Tpi1/Uox/Npdc1/Bpgm/Dut/Prkag 2/ Prkaca/Actn3/Pklr/Pgam 1/Ads l	20
MF	GO:001 6853	Isomerase activity	20/829	129/ 14076	6.36E+09	0.0435513 49247743 3	Ppil6/Aloxe3/Fkbp1b/Fkbp4/LOC500959/Trub2/Pin1/Gpi/Tpi1/Pmm1/Bpgm/Pdia2/Pgm2l1/Rpusd3/Fkbp8/Ptges 2/Ppie/Pgam1/Ppid/Ppih	20
BP	GO:000 6090	Pyruvate metabolic process	16/813	99/14671	0.0001020 78921163 352	0.0698459 77229872 5	Eno1/Gck/ENSRNOG00000063843/LOC500959/Gpi/Tpi1/Sds/Pdk2/Bpgm/Prkag2/Prkaca/Fahd1/Me3/Actn3/Pklr /Pgam1	16
BP	GO:004 6939	Nucleotide phosphorylation	14/813	84/14671	0.0001953 21682690 402	0.0698459 77229872 5	Nme5/Eno1/Gck/ENSRNOG00000063843/LOC500959/Nme1/Gpi/Tpi1/Bpgm/Prkag2/Prkaca/Actn3/Pklr/Pgam1	14
BP	GO:000 6165	Nucleoside diphosphate phosphorylation	14/813	84/14671	0.0001953 21682690 402	0.0698459 77229872 5	Nme5/Eno1/Gck/ENSRNOG00000063843/LOC500959/Nme1/Gpi/Tpi1/Bpgm/Prkag2/Prkaca/Actn3/Pklr/Pgam1	14
BP	GO:000 6094	Gluconeogenesis	11/813	52/14671	0.0001055 56501324 061	0.0698459 77229872 5	C1qtnf12/Pck2/Gck/G6pc3/Gpi/Sds/Stk11/Pdk2/Prkaca/Wdr5/Pgam1	11
BP	GO:001 9319	Hexose biosynthetic process	11/813	54/14671	0.0001511 63310344 414	0.0698459 77229872 5	C1qtnf12/Pck2/Gck/G6pc3/Gpi/Sds/Stk11/Pdk2/Prkaca/Wdr5/Pgam1	11

Cat.: Category; Descrip.: description; #: counts. Note: Category: Classification of GO databases, including biological processes (BP), cellularcomponents(CC), molecularfunction(MF).GOID: Unique identification id of Gene Ontology database. Description: Function description correspondingtothe GO number. GeneRatio: Ratio between the numberof differentially expressed genes in each GO term and all differentially expressed genes that can be found in GO database. BgRatio: In background GO database, the ratio of all genes concerning this GO term to all genes. *P* value: Statistics category term; abbreviation for probability value. *P*_adj_: Adjusted p-value. Generally, GO Terms with Corrected_*P* value <0.05 are significant enrichment. geneName: Differentially expressed genes in this term. Count: The numberof difference gene annotated to GO number.

**Figure 6:**
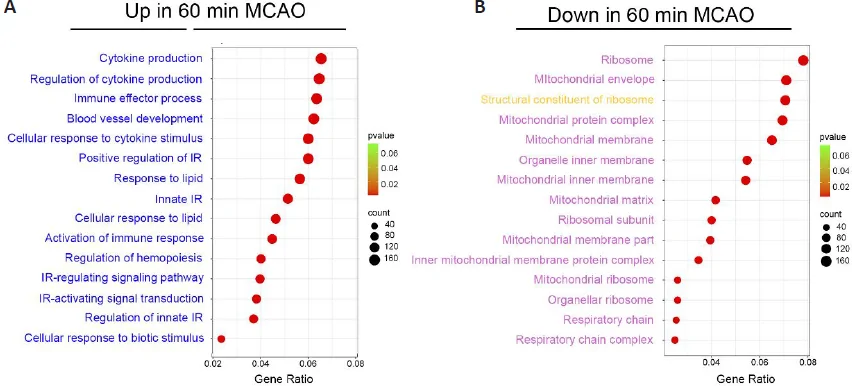
Gene Ontology (GO) enrichment analysis for the upregulated (A) and downregulated pathways (B) in the cerebral ipsilateral cortex after 15 days of reperfusion in moderate ischemia rats compared with sham rats. The y-axis shows the 15 most significant GO annotations (blue for biological processes, orange for molecular functions, and purple for cellular compartments). The x-axis indicates the ratio of the number of differentially expressed genes linked with the GO term to the total number of differentially expressed genes. The point size represents the number of genes annotated to a specific GO term, and the point color indicates the adjusted *P*-value. IR: Immune response; MCAO: middle cerebral artery occlusion.

GO analysis of the moderate ischemia versus the sham experimental groups revealed the immune response as the most significant upregulated biological process (**Figure 6A** and **Additional Table 3**). The top 15 downregulated GO terms are related to mitochondria, oxidative phosphorylation, and ribosome cellular compartments (**Figure 6B** and **Additional Table 4**).

**Additional Table 3:** Gene Ontology (GO) enrichment analysis for upregulated pathways in the cerebral ipsilateral cortex after 15 days of reperfusion in moderate ischemia rats compared with sham rats

Cat.	GOID	Descrip.	Gene ratio	Bg ratio	*P* value	*P*_adj_	geneName	#
BP	GO:00 01816	Cytokine production	168/2571	499/ 14572	1.22E-04	1.04E-01	F2r/Adcy7/Dennd1b/Col3a1/Rab7b/Cd84/Ptprc/Tlr3/Igf1/Lgr4/Runx1/Clec7a/Nlrp3/Thbs1/Dhx9/Oas2/Gpam/ Csf1r/Axl/Zbtb20/Nckap1l/C3ar1/Mapkbp1/N1rl1/Havcr2/Epha2/C3/Rig1/Arfgef2/Rora/Nlrc4/Tlr9/Nav3/Iqgap1/ Sa sh3/Rel/Unc93b1/Serpinb1a/Irf8/Slc11a1/Inpp5d/Gpnmb/Rsad2/Tmf1/Casp4/Lbp/Ccl3/Itgb8/Tlr7/Irgm/Nfkb1/ Nfam1/Kpna6/Lyn/Cx3cr1/Aim2/Card11/Smad4/Pde4d/Cd81/Pml/Tia1/Btk/Setd2/Tlr8/Tyk2/Stat3/Cd74/S1pr3/ Psen1/Cgas/Il18/Tnfsf4/Elf1/Erbin/Lum/Ptpn6/Oas3/Adamts3/Dhx58/Creb1/Irf7/Ifih1/Cmklr1/Hgf/Gbp5/Traf6/ Map3k7/N1r1/Bd3/Pld4/Malt1/Irf5/Cyp1b1/Wnt5a/Sting1/Tgfb1/Elf4/Nfatc4/Pmp/Trem2/Syk/Ankrd17/Rbm47 /Usp15/Gpr18/Cd14/Fgr/Mertk/N18r1/Tmed10/Itgav/Zfp36/Ticam2/Tlr6/Hif1a/Kit/Ssc5d/Clec5a/Casp1/Mapk9/ Bmpr1a/Abl1/Xiap/Lag3/Cd274/Appl1/Cyba/Tsku/Traf3/N4bp1/Pde4b/Pum 1/Tlr2/Plin3/Ripk2/Chma7/Zc3h12a/ Cd34/Foxp1/ Cd2/Slc37a4/Brca1/Abcd2/Lacc1/Appl2/Irak1/Jak2/Dhx33/Lpl/Adam17/Plcg2/Scamp5/Tnfrsf1b/Rasgrp1/Btn2a2/ Casp8/Sptbn1/AABR07030823.1/Vsig4/Dhx36/Tlr4/Pik3r1/Gsdmd/Ddx21/Chuk/Zcchc3/Atp2b1	168
BP	GO:00 01817	Regulation of cytokine production	166/2571	489/ 14572	7.89E-05	8.02E-02	F2r/Adcy7/Dennd1b/Rab7b/Cd84/Ptprc/Tlr3/Igf1/Lgr4/Runx1/Clec7a/Nlrp3/Thbs1/Dhx9/Oas2/Gpam/Csf1r/Axl/ Zbtb20/Nckap1l/C3ar1/Mapkbp1/N1rl1/Havcr2/Epha2/C3/Rig1/Arfgef2/Rora/Nlrc4/Tlr9/Nav3/Iqgap1/Sash3/ Rel/Unc93b1/Serpinb1a/Irf8/Slc11a1/Inpp5d/Gpnmb/Rsad2/Tmf1/Casp4/Lbp/Cd3/Itgb8/Tlr7/Irgm/Nfkb1/Nfam1/ Kpna6/Lyn/Cx3cr1/Aim2/Card11/Smad4/Pde4d/Cd81/Pml/Tia1/Btk/Setd2/Tlr8/Tyk2/Stat3/Cd74/S1pr3/Psen1/ Cgas/N18/Tnfsf4/Elf1/Erbin/Lum/Ptpn6/Oas3/Dhx58/Creb1/Irf7/Ifih1/Cmklr1/Hgf/Gbp5/Traf6/Map3k7/N1r1/Bd3 /Pld4/Malt1/Irf5/Cyp1b1/Wnt5a/Sting1/Tgfb1/Elf4/Nfatc4/Pmp/Trem2/Syk/Ankrd17/Rbm47/Usp15/Gpr18/Cd1 4/Fgr/Mertk/N18r1/Tmed10/Itgav/Zfp36/Ticam2/Tlr6/Hif1a/Kit/Ssc5d/Clec5a/Casp1/Mapk9/Bmpr1a/Abl1/Xiap/ Lag3/Cd274/Appl1/Cyba/Tsku/Traf3/N4bp1/Pde4b/Pum1/Tlr2/Plin3/Ripk2/Chma7/Zc3h12a/Cd34/Foxp1/Cd2/ Slc37a4/Brca1/Abcd2/Lacc1/Appl2/Irak1/Jak2/Dhx33/Lpl/Adam17/Plcg2/Scamp5/Tnfrsf1b/Rasgrp1/Btn2a2/Casp8/Sptbn1/ AABR07030823.1/Vsig4/Dhx36/Tlr4/Pik3r1/Gsdmd/Ddx21/Chuk/Zcchc3/Atp2b1	166
BP	GO:00 02252	Immune effector process	163/2571	471/ 14572	1.94E-05	3.29E-02	Kmt2a/Rif1/Rc3h1/Dennd1b/Cd84/Itgal/Ptprc/Tlr3/Kdm5d/Dock2/Parp9/Nfkbiz/Kmt2e/Nlrp3/Cd180/Gpam/Dhx15/Axl/ Nckap1l/Havcr2/C3/Rig1/Itgam/Dtx3l/Rora/Tlr9/Gbp2/Sash3/Trim56/Entpd7/Gbp7/Rac2/Vav1/Unc93b1 /Irf8/Slc11a1/Tsc1/Atad5/Inpp5d/Rsad2/Casp4/Lbp/Slfn2/Tlr7/Irgm/Ctsc/Lyn/Cx3cr1/Aim2/C4a/Cd81/Mlkl/ Eif2ak2/Btk/Setd2/Apbb1ip/Tlr8/Itgax/Stat3/Dock11/Gpr183/Ap1g1/Psen1/Cgas/Il18/Tnfsf4/Abcc9/Enpp3/Ptpn6/ Oas3/Phf14/Cfb/Dhx58/Fgl2/Tcirg1/Irf7/Was/Ifih1/Nlrc5/RT1-Da/Traf6/Map3k7/Il1r1/Bd3/Malt1/Irf5/Myo1g/ Wnt5a/Sting1/Adora3/Serping1/Pik3r6/Samhd1/Trem2/Fes/Syk/Spi1/Ankrd17/Usp15/Dock10/Fgr/Znfx1/Serpinb9/Anxa3/Gm/ Il18r1/Zbtb1/C8g/Prkce/AABR07028902.1/Lcp1/Ticam2/Cxadr/Kit/Casp1/Rnasel/Stat2/Zmpste24/Abl1/Lag3/Appl1/Traf3/ Snap23/Pum1/Tlr2/Mtor/Nsd2/Ripk2/Tp53/A2m/Swap70/Zc3h12a/Cd2/Ada/Irf2/Lig4/Cfd/C1qc/Ifitm2/Lacc1/ Appl2/Plec/Bcr/Plcg2/Fer/Tnfrsf1b/Bst2/Sin3a/Tfrc/Rasgrp1/C1qa/Stxbp2/Itch/AABR07030823.1/Dhx36/N7r/Tlr4/ RT1-DMb/Pla2g5/Notch2/Pdpk1/Ddx21/Zcchc3	163
BP	GO:00 01568	Blood vessel development	160/2571	497/ 14572	8.75E-02	3.71E+01	Col1a2/Tjp1/Col3a1/Ndnf/Adgra2/Col1a1/Tlr3/Pde3b/Pik3cg/Ptprm/Notch1/Tgfbr1/Prrx1/Dync2h1/Thbs1/ Pik3c2a/Nf1/Vash2/B4galt1/Arid2/Gli3/Erap1/Wasf2/Notch3/Mmp2/C3ar1/Mecp2/C1galt1/Hk2/Epha2/Ubp1/ Aldh1a2/C3/Cdh2/Rora/Cntrl/Bmpr2/Amot/Gpc3/Thbs2/Gna13/Calcrl/Flna/Tal1/Hmox1/Prdm1/Rock1/Itgb8/Plxnd1/ Bmp7/Prkca/Col4a3/Minar1/Wnk1/Shc1/Dcn/Stab1/Pml/Fzd4/Setd2/Foxc2/Sp1/Itgax/Stat3/Cxcl 10/Psen1/Il18/ Pik3cb/Lepr/Qki/Fgf2/Ago2/Rapgef1/Sema6a/Ntrk2/Socs3/Tgfbr2/Hectd1/Prkx/Sos1/Foxo1/Cyp1b1/Slc1a1/ Wnt5a/Pkd2/Tgfb1/Tie1/Ndst1/Parva/Pik3r6/Sash1/Nfatc4/Cd40/Syk/Spi1/Ankrd17/Angpt2/Mtdh/Prox1/Mia3/Ets1/ Loxl2/Anxa3/Adgrf5/Shh/Itgav/Emcn/Hif1a/Slit2/Hey2/Pknox1/Bmpr1a/Spint1/Abl1/Stard13/Sufu/Zfp36l1/Sox4 /Elk3/Gja1/Cdh5/Sgpl1/Efnb2/Creb3l1/Lama1/Zc3h12a/Smo/Cd34/Med1/Klf5/Ccl2/Brca1/Rasa1/Ednra/Tcf7l2/ Hpgd/Cul7/Myo1e/Nr2f2/Antxr1/Yap1/Nrp2/Angpt4/Emilin1/Lrp5/Itga5/Col5a1/Itgb1/Sec24b/E2f7/Dll4/Reck/ Pofut1/Notch2/Pdpk1/Rin2/Jak1/Npr2/Naglu/Lama4	160
BP	GO:00 71345	Cellular response to cytokine stimulus	154/2571	471/ 14572	6.82E-03	3.15E+01	Otud4/Rif1/Rab7b/Dock8/Notch1/Lifr/Parp9/Nfkbiz/Adamts12/Dhx9/Oas2/Arid5b/Csf1r/Csf2rb/Axl/Il1rl1/Hk2/ Cacnb4/Rora/Il1m/Trim25/Gbp2/Trim56/Ro60/Gbp7/Irf8/Ghr/Ythdf3/Sh2b3/Lrch1/Casp4/Adam23/Parp14/ Irg m/Nfkb1/Lyn/Eprs1/Aim2/Smad4/Wnk1/Hds1/Pml/Btk/Fzd4/Tnfrsf1a/Gbp4/Tyk2/Rps6ka5/Stat3/Cd74/Cxd10/ Gfpt2/Il18/Rab43/Cxd 11/Card14/Lepr/Ifit1/Erbin/Myo1c/Oas3/Sfrp1/Creb1/Tcirg1/Irf7/Was/Socs3/Ifih1/Nlrc5/ Gbp5/Traf6/Map3k7/Il1r1/Eps8/Ifngr1/Irf5/Ptprt/Slc1a1/Wnt5a/Aqp4/Sting1/Zbp1/Oasl/Samhd1/Dpysl3/Mcf2/ Trem2/Tnfsf13b/Syk/Spi1/Camk2a/Rbm47/Il6st/Mme/Kdm5b/N18r1/Ccdc3/LOC102556096/Dok1/Ikbkb/Sh2b2/ Zfp36/Adar/Tnfrsf13c/Ticam2/Stxbp4/Hif1a/Tpr/Kit/Slit2/Zfp36l2/Adamts7/Stat2/Xiap/Gldc/Appl1/Traf3/Zfp36l 1/Nfat5/Tlr2/Slc2a4/Ripk2/Tp53/Zc3h12a/Med1/Klf5/Abcg4/Ccl2/Brca1/Nr1d1/Pdia3/Appl2/Irak1/Jak2/Pde12/ Rarg/Adam17/Shpk/Yap1/Fer/Nrp2/Vim/Tfrc/Osmr/Piga/Mtf2/Pafah1b1/Sox9/Tlr4/Nfe2l2/ENSRNOG00000071 219/Jak1/Npr2/Chuk	154
BP	GO:00 50778	Positive regulation of immune response	154/2571	417/ 14572	1.54E-07	7.84E-05	Alpk1/Otud4/Rif1/Rc3h1/Dennd1b/Wdfy1/Rab7b/Nfatc2/Cblb/Ptprc/Tlr3/Lgr4/Parp9/Nfkbiz/Tlr13/Nlrp3/Pik3a p1/Nckap1l/C3ar1/Havcr2/C3/Rig1/Itgam/Tasl/Cacnb4/Tlr9/Trim25/Gbp2/Sash3/Trim56/Gbp7/Vav1/Unc93b1/Slc11a 1/Atad5/Rsad2/Casp4/Lbp/Tlr7/Irgm/Nfkb1/Nfam1/Lyn/Aim2/Card11/C4a/Pde4d/Cd81/Wnk1/Kcnn4/Btk/ Gbp4/Tlr8/Epg5/Cd38/Lcp2/Ap1g1/Psen1/Cgas/Il18/Tnfsf4/Elf1/Erbin/Ptpn6/Themis2/Oas3/Cfb/Dhx58/Klhl6/Irf 7/Ifih1/Nlrc5/Slc15a3/RT1-Da/Gbp5/Traf6/Map3k7/Il1r1/Sos1/Malt1/ Irf5/Myo1g/Wnt5a/Sting1/Zbp1/Adora3/Serping1/Prn p/Trem2/Tnfsf13b/Syk/Spi1/Braf/Ankrd17/Rbm47/Usp15/Cd14/Fgr/Znfx1/ Cd22/Il18r1/Zbtb1/LOC102556096/C8g/Cmtm3/Prk ce/Sh2b2/Mfhas1/Ticam2/Tlr6/Kit/Casp1/Ap3b1/Mapkapk3/Abl1/ Xiap/Lag3/Cd274/Appl1/Cyba/Traf3/Pde4b/P um1/Tlr2/Plin3/Nsd2/Ripk2/A2m/Blnk/Zc3h12a/Med1/Foxp1/Ada/Cfd/Nr1d1/C1qc/Lacc1/Appl2/Irak1/Plcg2/Cd8a/Sin3a/Tfrc/Rasgrp1/C1qa/Btn2a2/Dhx36/Tlr4/ RT1-DMb/Pdpk1/LOC681325/Naglu/Ddx21/Zcchc3	154
BP	GO:00 33993	Response to lipid	145/2571	416/ 14572	1.01E-03	7.36E-01	Atm/F2r/Abca 1/Lats1/Setx/Spp1/Tgfbr1/Ubr5/Plaa/Trim24/Nfkbiz/Tgfbr3/Nlrp3/Ncoa2/Cd180/Lcor/Tbl1x/Axl/A bcb4/Ncoa3/Mecp2/Havcr2/Asxl1/Aldh1a2/Gramd1c/Prkaa2/Rora/N1rn/Gsk3b/Rest/Gbp2/Abl2/Irf8/Prkag1/ Slc11a 1/Gramd1b/Epha3/Tmf1/Casp4/Lbp/Ar/Nfkb1/Foxo3/Lrp6/Lyn/Cx3cr1/Ppargc1b/Ptgfr/Bmp7/Prkca/Pde4d/ Gbp4/Fam210b/Stat3/Ptafr/Cxcl 10/Abhd2/Pim1/Tifab/Il18/Esr2/Erbin/Ppm1e/P2ry6/Ka nk2/Sfrp1/Creb1/Dab2/ Cdc73/Tgfbr2/Gbp5/Traf6/Malt1/Ly86/Adam9/Foxo1/Wnt5a/Tie1/Sash1/Nr3c1/Trem2/Fes/Syk/Spi1/Mtdh/ Cd1 4/Arid1a/Ntrk3/Ncor1/Pgr/Nrip1/Prkce/Prkaa1/Zfp36/Slit2/Zfp36l2/Casp1/Mapkapk3/Abl1/Zfp36l1/Cnot1/Zbtb 7a/Cyp7b1/Pde4b/Fbxo32/Ripk2/Adcy6/Klf9/Zc3h12a/Smo/Trib1/Med1/Foxp1/Ccl2/Brca1/Nr1d1/Irak1/Jak2/ Hadhb/Bcr/Lpl/Rarg/Adam17/Shpk/Plcg2/Yap1/Acsbg1/Trip4/Fer/Tnfrsf1b/Npc1/ Vim/Rxra/Scnn1a/Ccl19/Per1/Atp1a2/Sox9/Ube3a/Tlr4/Clock/Olr59/Naglu/Nedd4/Brinp3	145
BP	GO:00 45087	Innate immune response	132/2571	370/ 14572	3.57E-03	2.24E+00	Alpk1/Otud4/Wdfy1/Rab7b/Tlr3/Lgr4/Parp9/Nfkbiz/Tlr13/Pik3ap1/Havcr2/Nlrp1a/Rig1/Tasl/Tlr9/Ch25h/Trim25 /Gbp2/Trim56/Gbp7/Vav1/Unc93b1/Serpinb1a/Cybb/Irf8/Slc11a1/Ythdf3/Cxcl16/Rsad2/Lbp/Parp14/Tlr7/Irgm/Lyn/Cx3cr1/ Eprs1/Aim2/Trim21/Pml/Setd2/Gbp4/Tlr8/Tyk2/Epg5/Cd74/Ap1g1/Cgas/Il18/Rab43/Lrp8/Erbin/Ptpn6/ Myo1c/Oas3/Uba7/Dhx58/Irf7/Was/Ifih1/Nlrc5/Slc15a3/Gbp5/Ifngr1/Malt1/Irf5/Wnt5a/Aqp4/Sting1/Zbp1/ Serping1/Pik3r6/Samhd1/Trem2/RT1-Ba/Mid2/Syk/Spi1/Camk2a/Ankrd17/Rbm47/ Usp15/Cd14/Fgr/Znfx1/Parp1/Grn/LOC102556096/Prkce/Mfhas1/ AABR07028902.1/Adar/Ticam2/Stxbp4/Tlr6/Clec5a/Ap3b1/Mapkapk3/Trim26/Stat2/Xiap/Lag3/Appl1/Cyba/Traf3/N4bp1/Pum1/Tlr2/ Plin3/Ripk2/Tp53/A2m/Med1/Ccl2/Cfd/Nr1d1/Lacc1/Appl2/Irak1/Pde12/Plcg2/Vim/Sin3a/ Rasgrp1/Slc22a5/Tlr4/Nfe2l2/Pdpk1/Jak1/LOC681325/Naglu/Nedd4/Zcchc3	132
BP	GO:00 71396	Cellular responseto lipid	119/2571	299/ 14572	6.85E-07	1.74E-02	Atm/Abca 1/Lats1/Setx/Spp1/Ubr5/Plaa/Trim24/Nfkbiz/Nlrp3/Cd180/Lcor/Axl/Abcb4/Ncoa3/Havcr2/Aldh1a2/ Gramd1c/Prkaa2/Rora/Gsk3b/Rest/Gbp2/Abl2/Irf8/Prkag1/Gramd1b/Epha3/Tmf1/Lbp/Ar/Nfkb1/Lrp6/Lyn/Cx3cr1 /Ppargc1b/Ptgfr/Bmp7/Prkca/Pde4d/Gbp4/Fam210b/Cxcl 10/Abhd2/Pim1/Tifab/Il18/Esr2/Ppm1e/P2ry6/Ka nk2/ Sfrp1/Creb1/Dab2/Cdc73/Gbp5/Traf6/Malt1/Ly86/Adam9/Wnt5a/Sash1/Nr3c1/Trem2/Fes/Syk/Spi1/Mtdh/ Cd14/Arid1a/Ntrk3/Ncor1/Pgr/Nrip1/Prkce/Prkaa1/Zfp36/Zfp36l2/Abl1/Zfp36l1/Cnot1/Zbtb7a/Cyp7b1/Pde4b/ Fbxo32/Ripk2/Adcy6/Klf9/Zc3h12a/Smo/Trib1/Med1/Foxp1/Ccl2/Brca1/Nr1d1/Irak1/Jak2/Hadhb/Bcr/Lpl/Shpk/Plcg2 /Yap1/Trip4/Tnfrsf1b/N pc1/Vim/Rxra/Scnn1a/Per1/Atp1a2/Sox9/Ube3a/Tlr4/Clock/Olr59/Nedd4/Brinp3	119
BP	GO:00 02253	Activation of immune response	115/2571	291/ 14572	5.58E-05	7.09E-02	Alpk1/Otud4/Rc3h1/Dennd1b/Wdfy1/Rab7b/Nfatc2/Cblb/Ptprc/Tlr3/Lgr4/Nfkbiz/Tlr13/Pik3ap1/Nckap1l/C3ar1/ Havcr2/C3/Rig1/Tasl/Cacnb4/Tlr9/Trim25/Gbp2/Gbp7/Unc93b1/Rsad2/Lbp/Tlr7/Nfkb1/Nfam1/Lyn/Aim2/Card1 1/C4a/Pde4d/Cd81/Wnk1/Kcnn4/Btk/Gbp4/Tlr8/Epg5/Cd38/Lcp2/Psen1/Cgas/Elf1/Erbin/Ptpn6/Themis2/Oas3/ Cfb/Dhx58/Klhl6/Irf7/Ifih1/Slc15a3/Gbp5/Traf6/Sos1/Malt1/Myo1g/Sting1/Serping1/Prnp/Trem2/Syk/Braf/ Ankrd17/Usp15/Cd14/Znfx1/Cd22/LOC102556096/C8g/Cmtm3/Prkce/Sh2b2/Mfhas1/Ticam2/Tlr6/Ap3b1/Mapkapk3 /Abl1/Xiap/Appl1/Cyba/Traf3/Pde4b/Pum1/Tlr2/Plin3/Ripk2/A2m/Blnk/Zc3h12a/Foxp1/Ada/Cfd/Nr1d1/C1qc/Lacc1/ Appl2/Irak1/Plcg2/Cd8a/Sin3a/C1qa/Btn2a2/Tlr4/Pdpk1/LOC681325/Naglu/Zcchc3	115
BP	GO:19 03706	Regulation of hemopoiesis	103/2571	274/ 14572	2.15E-01	7.27E+01	Rc3h1/Rab7b/Fanca/Acvr2a/Notch1/Runx1/Nfkbiz/Nlrp3/Nf1/Gli3/Axl/Nckap1l/Tlr9/Sash3/Evi2b/Inpp5d/Fnip1/Tal 1/Prdm1/Atp11c/Irgm/Mmp14/Nfam1/Lyn/Ppargc1b/Card11/Itpkb/Hcls1/Csf3r/Fam210b/Stat3/Cd74/Setd1 a/Fbn1/Pim1/Il18/Tnfsf4/Tmem131l/Mafb/Ptpn6/Sos2/Fgl2/Sfrp1/Creb1/Tmem176a/Cdc73/Tgfbr2/RT1- Da/Traf6/Sos1/Malt1/Wnt5a/Tgfb1/Pik3r6/Faxdc2/Trem2/Fes/RT1- Ba/Prkdc/Syk/Spi1/Braf/Ets1/Zeb1/Sox13/Zbtb1/Shh/Zfp36/Mitf/Hif1a/Zfp36l2/Ap3b1/Abl1/Rcor1/Lag3/Zfp36l1 /Sox4/Mtor/Ripk2/Zc3h12a/Trib1/Med1/Foxp1/Ada/Klf13/C1qc/Abcb10/Rarg/Inhba/Rasgrp1/Tnfaip6/Btn2a2/C asp8/AABR07030823.1/Dhx36/Cdk6/Il7r/Rhoh/Nfe2l2/Atxn1l/ENSRNOG00000071219/Notch2/Pik3r1	103
BP	GO:00 02764	Immune response regulating signaling pathway	102/2571	257/ 14572	3.96E-03	2.24E+00	Alpk1/Otud4/Rc3h1/Dennd1b/Wdfy1/Rab7b/Nfatc2/Cblb/Ptprc/Tlr3/Lgr4/Nfkbiz/Tlr13/Pik3ap1/Nckap1l/C3ar1/ Havcr2/Lat2/Rig1/Tasl/Cacnb4/Tlr9/Trim25/Unc93b1/Rsad2/Lbp/Tlr7/Nfkb1/Nfam1/Lyn/Card11/Pde4d/Cd81/ Wnk1/Kcnn4/Btk/Tlr8/Epg5/Cd38/Lcp2/Psen1/Cgas/Elf1/Erbin/Ptpn6/Themis2/Oas3/Dhx58/Klhl6/Irf7/fih1/ Slc15a3/Traf6/Sos1/Malt1/Myo1g/Sting1/Prnp/Trem2/Syk/Braf/Ankrd17/Usp15/Cd14/Cd22/Cmtm3/Prkce/Sh2b2/ Ctsh/Mfhas1/Ticam2/Tlr6/Kit/Ap3b1/Mapkapk3/Abl1/Xiap/Appl1/Cyba/Traf3/Pde4b/Pum1/Tlr2/Plin3/Ripk2/Blnk/ Zc3h12a/Foxp1/Ada/Nr1d1/Lacc1/Appl2/Irak1/Plcg2/Cd8a/Fer/Btn2a2/Tlr4/Pdpk1/LOC681325/Naglu/Zcchc3	102
BP	GO:00 02757	Immune response activating signal transduction	98/2571	248/ 14572	2.32E-02	1.18E+01	Alpk1/Otud4/Rc3h1/Dennd1b/Wdfy1/Rab7b/Nfatc2/Cblb/Ptprc/Tlr3/Lgr4/Nfkbiz/Tlr13/Pik3ap1/Nckap1l/C3ar1/ Havcr2/Rig1/Tasl/Cacnb4/Tlr9/Trim25/Unc93b1/Rsad2/Lbp/Tlr7/Nfkb1/Nfam1/Lyn/Card11/Pde4d/Cd81/Wnk1/ Kcnn4/Btk/Tlr8/Epg5/Cd38/Lcp2/Psen1/Cgas/Elf1/Erbin/Ptpn6/Themis2/Oas3/Dhx58/Klhl6/Irf7/Ifih1/Slc15a3/ Traf6/Sos1/Malt1/Myo1g/Sting1/Prnp/Trem2/Syk/Braf/Ankrd17/Usp15/Cd14/Cd22/Cmtm3/Prkce/Sh2b2/Mfhas1/ Ticam2/Tlr6/Ap3b1/Mapkapk3/Abl1/Xiap/Appl1/Cyba/Traf3/Pde4b/Pum1/Tlr2/Plin3/Ripk2/Blnk/Zc3h12a/ Foxp1/Ada/Nr1d1/Lacc1/Appl2/Irak1/Plcg2/Cd8a/Btn2a2/Tlr4/Pdpk1/LOC681325/Naglu/Zcchc3	98
BP	GO:00 45088	Regulation of innate immune response	95/2571	243/ 14572	1.50E-01	5.88E+01	Alpk1/Otud4/Wdfy1/Rab7b/Tlr3/Lgr4/Parp9/Nfkbiz/Tlr13/Pik3ap1/Havcr2/Rig1/Tasl/Tlr9/Trim25/Gbp2/Trim56/ Gbp7/Vav1/Unc93b1/Serpinb1a/Ythdf3/Rsad2/Lbp/Parp14/Tlr7/Irgm/Lyn/Aim2/Trim21/Gbp4/Tlr8/Epg5/Ap1g1 /Cgas/Lrp8/Erbin/Oas3/Dhx58/Irf7/Ifih1/Nlrc5/Slc15a3/Gbp5/Wnt5a/Sting1/Zbp1/Serping1/Pik3r6/Samhd1/Tre m2/Syk/Spi1/Ankrd17/Rbm47/Usp15/Cd14/Fgr/Znfx1/Parp1/Grn/LOC102556096/Prkce/Mfhas1/Adar/Ticam2/Tl r6/Ap3b1/Mapkapk3/Stat2/Xiap/Lag3/Appl1/Cyba/Traf3/N4bp1/Pum1/Tlr2/Plin3/Ripk2/A2m/Med1/Nr1d1/Lacc 1/Appl 2/Irak1/Plcg2/Sin3a/Rasgrp1/Tlr4/Nfe2l2/Pdpk1/LOC681325/Naglu/Zcchc3	95
BP	GO:00 71216	Cellular responseto biotic stimulus	60/2571	123/ 14572	2.03E-01	7.27E+01	Abca 1/Notch1/Plaa/Clec7a/Nfkbiz/Nlrp3/Cd180/Axl/Havcr2/Gsk3b/Gbp2/Irf8/Lbp/Nfkb1/Lyn/Cx3cr1/Prkca/ Pde4d/Gbp4/Cxcl10/Tifab/Il18/Ppm1e/Cdc73/Gbp5/Traf6/Malt1/Ly86/Adam9/Wnt5a/Sash1/Trem2/Syk/Spi1/Mtdh /Cd14/Prkce/Zfp36/Tlr6/Zmpste24/Abl1/Pde4b/Tlr2/Ripk2/Tp53/Zc3h12a/Trib1/Ccl2/Nr1d1/Irak1/Jak2/Hadhb/ Bcr/Shpk/Plcg2/Tnfrsf1b/Vim/Tlr4/Ppp1r15b/Notch2	60

Cat.: category; Descrip.: description; #: counts. Note: Category: Classification of GO databases, including biological processes (BP), cellularcomponents(CC), molecularfunction(MF).GOID: Unique identification id of Gene Ontology database. Description: Function description correspondingtothe GO number. GeneRatio: Ratio between the numberof differentially expressed genes in each GO term and all differentially expressed genes that can be found in GO database. BgRatio: In background GO database, the ratio of all genes concerning this GO term to all genes. *P* value: Statistics category term; abbreviation for probability value. *P*_adj_: Adjusted p-value. Generally, GO Terms with Corrected_P value <0.05 are significant enrichment. geneName: Differentially expressed genes in this term. Count: The numberof difference gene annotated to GO number.

**Additional Table 4:** Gene Ontology (GO) enrichment analysis for downregulated pathways in the cerebral ipsilateral cortex after 15 days of reperfusion in moderate ischemia rats compared with sham rats

Cat.	GOID	Descrip.	Gene ratio	Bg ratio	*P* value	*P*_adj_	geneName	#
CC	GO:00 05840	Ribosome	144/1844	471/ 13385	6.57E-08	2.90E-06	Mrpl 28/Rpl34-ps 1/M rpl27/Rps14/M rpl37/M rpl3/M rpl44/LOC100362366/M rps 14/M rps6/Mtg2/Rpl22l1/M rpl34/U ba52/ Rpl8- ps7/Chchd1/Mrpl4/Rpl30l2/Oscp1/Mrpl12/Mrpl33/Mrps26/Mrpl55/LOC102555453/Rps21/Rps23/Mrps12/ Rpl9- ps30/Mpv17l2/Rps3/Rps19/AABR07049516.1/Rpl37/Mrpl14/Rpl35a/M rpl21/LOC687780/M rpl46/AABR07068536.1/ LOC100365810/Rpl 31/Rps21-ps1/Mrpl18/Zcchc17/Mrpl20/ENSRNOG00000063418/Rps29/Mrpl35/Mrpl54/Fau/Mrpl17/ Mrps18c/Rpl32- ps1/Rpl27/Rpl10a/Rpsa/Mrpl2/Rps27a-ps 1/Rplp2/Mrpl49/Rps16/Mrps2/Mrps18a/Rps13/Rpl28/Mrps16/ Rpl35/Mrpl41/Rpl4-ps 1/Rpl18/Rps7/Mrps10/Rpl29/Rps15/Mrps23/LOC100365839/Mrps27/Mrps7/Rps11/LOC108348870/ Rps20- ps10/Rps13/mrpl24/Mrpl48/AABR07072207.1/Rpl34/Mrpl15/Rpl13a/LOC100359505/Mrpl19/Mrpl58/Rpl13/Pelo/ Rpl 22/Rpsa-ps4/Mrpl13/Rpl18a/Rps8/LOC100125366/RGD1359290/Rplp1/Rps28/Rps3a/ LOC100363452/Rsl24d1/Rps9/ENSR NOG00000066953/Mrps15/Mrpl30/ENSRNOG00000067510/ Rpl17/Mrps9/Rps27/mrpl9/Mrps21/LOC103690996/Mrps18b/Mrpl23/ Rpl26/Rpl23/Rpl32/mrpl11/ENSRNOG00000068816/LOC108349446/Rpl11/LOC120097744/LOC103694242/ Rpl15/Rpl19/Rpl21/AC135389.1/Rpl22/LOC100364191/Mrpl16/Mrpl57/Mrpl43/Rpl36a/Mrpl36/ Hspa 14/AABR07030162.1/LOC498555/Rpl37l1/Ikzf4/ENSRNOG00000069856	144
CC	GO:00 05740	Mitochondrial envelope	131/1844	372/ 13385	2.58E-12	2.12E-10	Tmem160/Arl2/Samm50/Tmem11/Tomm40/Ndufb8/Chchd6/Mtln/Ndufa8/Ndufb2/Ndufs6l1/Sirt5/Fis1/Ndufb6 /Rab5if/Timm22/Uqcc4/Ndufv1/Uqcrq/Cyc1/Cox6a1/Fkbp8/Mtg2/Rhot2/Atp5f1d/Smdt1/Ndufc1/Bad/Ndufaf3/ Mt- co3/Uqcrc1/Cd320/Rmdn3/Ndufs7/Cox7a2/Pgam5/Timm17a/Atp5me/Apoo/Atp5mg/Mpv17l2/Coa4/Ndufc2/Mt fp1/Rcc1l/Chchd10/Tmem223/Slc25a11/Coq2/Uqcrh/Cox7c/Ndufa4/Ndufv3/Atp5f1b/Cox7a2l/Cox4i1/Tomm20/ Rexo2/Atp5pb/Timm21/Fech/Tomm22/Timm10/Pam16/Slc25a14/Uqcr10/Cox5b/Slc25a4/Coq7/Ndufa12/Agpat 4/Mpc2/Myoc/Atp5mk/Atp5po/Ndufa3-ps3/Vdac3/Mt-cyb/Bnip1/Ndufs4/Wasf1/Stoml2/Phb2/Coq9/ Slc25a5/Ndufa6/Ndufb5/Gatm/Ndufa3/Sdhd/Timm23/Mff/Sphk2/Cox5a/Triap1/Atp5pf/ Vdac1/Tmem177/Dmac2/Ndufa11/Uqcc1/Coq5/Ndufab1/Atp5f1e/Atp5pd/Ndufb10/Chc hd3/Tomm7/Mt- co2/Bok/Slc25a22/Prelid3b/Timm8a1/Pld6/Mtx1/Uqcrb/Ndufs3/Ndufaf5/Ptrti2/Htra2/Trmt10b/Ndufs8/Slc25a42/Bd2l 2/Atp5mk-ps2/Mt-co1/Ndufa9/Pmpca/Slc25a31/Tomm5/Coq3	131
MF	GO:00 03735	Structural constituent of ribosome	130/1840	429/ 13978	3.30E-08	2.62E-04	M rps 34/M rpl 28/Rpl34-ps 1/Mrpl27/Mrps25/Rps14/M rpl37/M rpl3/LOC100362366/ Mrps14/M rps6/Rpl22l1/M rpl34/U ba52/Rpl8-ps7/M rpl4/Rpl30l2/Oscp1/M rpl12/M rpl33/M rpl55/ LOC102555453/Rps21/Rps23/Mrps12/Rpl9-ps30/Rps3/Rps19/AABR07049516.1/ Rpl37/Mrpl14/Rpl35a/LOC687780/Mrpl46/AABR07068536.1/LOC100365810/Rpl31/Rps21-ps1/Mrpl18/ Mrpl20/ENSRN0G00000063418/Rps29/Mrpl35/Fau/Mrpl17/Mrps18c/Rpl32- ps1/Rpl27/Rpl10a/Rpsa/Mrpl2/ Rps27a-ps 1/Rplp2/Mrpl49/Rps16/Mrps2/Mrps18a/Rps13/Rpl28/Mrps16/Rpl35/Mrpl41/Rpl4-ps1/ Rpl18/Rps7/Rpl29/Rps15/Mrps23/LOC100365839/Rps11/LOC108348870/Rps20-ps 10/Rps13/ mrpl24/AABR07072207.1/Rpl34/Mrpl15/Rpl13a/LOC100359505/Mrpl19/Rpl13/Rpl22/Rpsa-ps4/ Mrpl13/Rpl18a/Rps8/LOC100125366/RGD1359290/Rplp1/Rps28/Rps3a/LOC100363452/Rsl24d1/Rps9/ ENSR N0G00000066953/Mrps 15/Mrpl 30/ENSRN0G00000067510/Rpl17/Mrps9/Rps27/mrpl9/Mrps21/L0C103690996/ Mrps18b/Mrpl23/Rpl26/Rpl23/Rpl32/mrpl11/LOC108349446/Rpl11/LOC120097744/LOC103694242/Rpl15/Rpl19/Rpl 21/AC135389.1/Rpl22/LOC100364191/Mrpl16/Mrpl57/Mrpl43/Rpl36a/Mrpl36/AABR07030162.1/LOC498 555/Rpl37l1/Ikzf4/ENSRN0G00000069856	130
CC	GO:00 98798	Mitochondrial protein complex	128/1844	222/ 13385	8.16E-40	4.67E-37	Mrpl28/Mrpl27/Samm50/Tomm40/Ndufb8/Chchd6/Ndufa8/Ndufb2/Ndufs6l1/Mrpl37/Mrpl3/Mrpl44/Ndufb6/ Timm22/Ndufv1/Uqcrq/Cyc1/Cox6a1/Mrps14/Mrps6/Idh3g/Mtg2/Atp5f1d/Smdt1/Suclg1/Chchd1/M rpl4/ Ndufc1/Mt-co3/Uqcrc1/Mrpl12/Ndufs7/Mrps26/Mrpl55/Cox7a2/Hsd17b10/Timm17a/ Atp5me/Apoo/Atp5mg/Mrps12/Mpv17l2/Mrpl14/Ndufc2/Chchd10/Mrpl21/Uqcrh/ Mrpl46/Cox7c/Ndufa4/Ndufv3/Atp5f1b/Cox7a2l/Mrpl18/Cox4i1/Mrpl20/Tomm20/ Mrpl35/Mrpl54/Mrpl17/Mrps18c/Atp5pb/Idh3b/Timm21/Tomm22/Timm10/Pam16/Uqcr10/ Mrpl2/Cox5b/Mrpl49/Slc25a4/Mrps18a/Mrps16/Bckdhb/Mrpl41/Ndufa12/Mrps10/Mrps27/Atp5mk/Atp5po/ Ndufa3-ps3/Mrps7/Ndufs4/Phb2/mrpl24/Mrpl48/Ndufa6/Ndufb5/Mrpl15/Ndufa3/Timm23/ Mrpl19/Mrps36/Cox5a/Mrpl58/Atp5pf/Mrpl13/Dmac2/Ndufa11/Ndufab1/Atp5f1e/Atp5pd/Ndufb10/Mrps15/ Chchd3/mrpl9/Mrps21/Mrps18b/Tomm7/Mt-co2/mrpl11/ENSRNOG00000068816/Timm8a1/Mtx1/Uqcrb/Ndufs3/Trmt10b/ Ndufs8/Atp5mk-ps 2/Mt-co1/Ndufa9/Mrpl16/Mrpl57/Mrpl43/Mrpl36/Slc25a31/Tomm5	128
CC	GO:00 31966	Mitochondrial membrane	120/1844	340/ 13385	2.84E-11	1.62E-08	Tmem160/Samm50/Tmem11/Tomm40/Ndufb8/Chchd6/Mtln/Ndufa8/Ndufb2/Ndufs6l1/Fis1/Ndufb6/Rab5if/ Ti mm22/Uqcc4/Ndufv1/Uqcrq/Cyc1/Cox6a1/Mtg2/Rhot2/Atp5f1d/Smdt1/Ndufc1/Bad/Ndufaf3/ Mt-co3/Uqcrc1/Cd320/Rmdn3/Ndufs7/Cox7a2/Pgam5/Timm17a/Atp5me/Apoo/Atp5mg/Mpv17l2/Ndufc2/Mtfp1/ Rcc1l/Chchd10/Tmem223/Slc25a11/Coq2/Uqcrh/Cox7c/Ndufa4/Ndufv3/Atp5f1b/Cox7a2l/Cox4i1/Tomm20/ Atp5pb/Timm21/Fech/Tomm22/Timm10/Pam16/Slc25a14/Uqcr10/Cox5b/Slc25a4/Coq7/Ndufa12/Agpat4/Mpc2/ Myoc/Atp5mk/Atp5po/Ndufa3-ps3/Vdac3/Mt-cyb/Bnip1/Ndufs4/Wasf1/Stoml2/Phb2/Coq9/Slc25a5/Ndufa6/Ndufb5/Ndufa3/ Timm23/Mff/Sphk2/Cox5a/Atp5 pf/Vdac1/Tmem177/Dmac2/Ndufa11/Uqcc1/Coq5/Ndufab1/Atp5f1e/Atp5pd/ Ndufb10/Chchd3/Tomm7/Mt- co2/Bok/Slc25a22/Pld6/Mtx1/Uqcrb/Ndufs3/Ndufaf5/Ptrh2/ Trmt10b/Ndufs8/Slc25a42/Bcl2l2/Atp5mk-ps2/Mt-co1/Ndufa9/Pmpca/Slc25a31/Tomm5/Coq3	120
CC	GO:00 19866	Organelle inner membrane	101/1844	253/ 13385	2.50E-11	1.59E-10	Tmem160/Tmem11/Tomm40/Ndufb8/Chchd6/Mtln/Ndufa8/Ndufb2/Ndufs6l1/Ndufb6/Rab5if/Timm22/Uqcc4/ Ndufv1/Uqcrq/Cyc1/Cox6a 1/Mtg2/Atp5f1d/Smdt1/Emd/Ndufc1/Ndufaf3/Mt-co3/Uqcrc1/Cd320/Ndufs7/Cox7a2/Pgam5/ Timm17a/Atp5me/Apoo/Atp5mg/Mpv17l2/Ndufc2/Mtfp1/Rcc1l/Chchd10/Tmem223/Slc25a11/Coq2/Uqcrh/Cox7c/Ndufa4/Nduf v3/Atp5f1b/Cox7a2l/Cox4i1/Atp5pb/Timm21/Fech/ Timm10/Pam16/Uqcr10/Cox5b/Slc25a4/Coq7/Ndufa12/ Mpc2/Myoc/Atp5mk/Atp5po/Ndufa3-ps3/Mt-cyb/Ndufs4/Stoml2/Phb2/Coq9/Slc25a5/Ndufa6/Ndufb5/ Ndufa3/Timm23/Sphk2/Cox5a/Atp5pf/Tmem177/Dma c2/Ndufa 11/Uqcc1/Coq5/Ndufab1/Atp5f1e/Atp5pd/Ndufb10/ Chchd3/Mt-co2/Bok/Slc25a22/Uqcrb/Ndufs3/Ndufaf5/Trmt10b/Ndufs8/Slc25a42/Atp5mk-ps2/ Mt-co1/Ndufa9/Pmpca/Slc25a31/Coq3	101
CC	GO:00 05743	Mitochondrial inner membrane	100/1844	227/ 13385	3.46E-15	9.93E-14	Tmem160/Tmem11/Tomm40/Ndufb8/Chchd6/Mtln/Ndufa8/Ndufb2/Ndufs6l1/Ndufb6/Rab5if/Timm22/Uqcc4/ Ndufv1/Uqcrq/Cyc1/Cox6a 1/Mtg2/Atp5f1d/Smdt1/Ndufc1/Ndufaf3/ Mt-co3/Uqcrc1/Cd320/Ndufs7/Cox7a2/Pgam5/Timm17a/Atp5me/Apoo/Atp5mg/Mpv17l2/Ndufc2/Mtfp1/Rcc1l/ Chc hd10/Tmem223/Slc25a11/Coq2/Uqcrh/Cox7c/Ndufa4/Ndufv3/Atp5f1b/Cox7a2l/Cox4i1/Atp5pb/Timm21/Fech/ Timm10/Pam16/Uqcr10/Cox5b/Slc25a4/Coq7/Ndufa12/Mpc2/Myoc/Atp5mk/Atp5po/Ndufa3-ps3/ Mt-cyb/Ndufs4/Stoml2/Phb2/Coq9/Slc25a5/Ndufa6/Ndufb5/Ndufa3/Timm23/Sphk2/Cox5a/Atp5pf/Tmem177/Dma c2/ Ndufa11/Uqcc1/Coq5/Ndufab1/Atp5f1e/Atp5pd/Ndufb10/Chchd3/Mt- co2/Bok/Slc25a22/Uqcrb/Ndufs3/Ndufaf5/Trmt10b/Ndufs8/Slc25a42/Atp5mk-ps2/Mt- co1/Ndufa9/Pmpca/Slc25a31/Coq3	100
CC	GO:00 05759	Mitochondrial matrix	77/1844	189/ 13385	3.22E-06	1.32E-04	Mrpl28/Mrpl 27/Grpel1/Alkbh7/Mrpl37/Sirt5/Mrpl3/Mrpl44/Mrps14/Mrps6/Idh3g/Mtg2/Hax1/Suclg1/Glrx5/ Dnaja 3/Chchd1/Mrpl4/Coasy/Mrpl12/Sirt3/Mrps26/Mrpl55/Hsd17b10/Mrps12/Mpv17l 2/Clpp/Mrpl14/Mrpl21/ Mrpl46/Atp5f1b/Mrpl18/Mrpl20/Rexo2/Mrpl35/Me3/Mrpl54/Mrpl17/Mrps18c/Idh3b/Mrpl2/Mrpl49/Mrps18a/ Mrps 16/Bckdhb/Mrpl41/Mrps10/Got2/Mrps27/Mrps7/Mdh2/mrpl24/Mrpl48/Mrpl15/Mrpl19/Mrps36/Mrpl58/ Mrpl13/Coq5/Mrps15/mrpl9/Mrps21/Mrps18b/Mlycd/Nudt1/mrpl11/ENSRNOG00000068816/Grsf1/Ddx28/Eral1/ Poldip2/Tufm/Ndufa9/Mrpl16/Mrpl57/Mrpl43/Mrpl36	77
CC	GO:00 44391	Ribosomal subunit	74/1844	150/ 13385	1.13E-11	8.06E-11	Mrpl28/Mrpl27/Mrpl37/Mrpl3/Mrpl44/Mrps14/Mrps6/Rpl8-ps7/Mrpl4/Rpl30l2/Mrpl12/Mrps26/Mrpl55/Rps23/Mrps12/Mpv17l 2/Rps3/Mrpl14/Mrpl21/Mrpl46/Mrpl18/Zcc hc17/Mrpl20/Rps29/Mrpl35/Mrpl54/Mrpl17/Mrps18c/Rpl27/ Rpl10a/Rpsa/Mrpl2/Rplp2/Mrpl49/Mrps2/Mrps18 a/Mrps16/Rpl35/Mrpl41/Mrps10/Rps15/Mrps27/Mrps7/Rps20-ps 10/mrpl24/Mrpl15/Rpl13a/Mrpl19/Mrpl58/Rpsa-ps4/Mrpl13/RGD1359290/Rps28/Rps9/ Mrps15/Mrpl30/Rpl17/Rps27/mrpl9/Mrps21/Mrps18b/Rpl26/mrpl11/ENSRNOG00000068816/LOC103694242/Rpl 15/Rpl19/Rpl22/LOC100364191/M rpl16/M rpl57/M rpl43/M rpl36/LOC4 98555	74
CC	GO:00 44455	Mitochondrial membrane part	73/1844	139/ 13385	1.49E-13	2.85E-11	Samm50/Tomm40/Ndufb8/Chchd6/Ndufa8/Ndufb2/Ndufs6l1/Ndufb6/Rab5if/Timm22/Ndufv1/Uqcrq/Cyc1/ Cox6a 1/Atp5f1d/Smdt1/Ndufc1/Mt-co3/Uqcrc1/Ndufs7/Cox7a2/Timm17a/Atp5me/Apoo/Atp5mg/Ndufc2/ Chchd10/Uqcrh/Cox7c/Ndufa4/Ndufv3/Atp5f1b/Cox7a2l/Cox4i1/Tomm20/Atp5pb/ Timm21/Tomm22/Timm10/Pam16/Uqcr10/Cox5b/Ndufa12/Atp5mk/Atp5po/Ndufa3-ps3/Ndufs4/Phb2/ Ndufa6/Ndufb5/Ndufa3/Timm23/Cox5a/Atp5pf/Dmac2/Ndufa11/Ndufab1/Atp5f1e/Atp5pd/ Ndufb10/Chchd3/Tomm7/ Mt-co2/Mtx1/Uqcrb/Ndufs3/Ndufaf5/Trmt10b/Ndufs8/Atp5mk-ps2/Mt-co1/Ndufa9/Tomm5	73
CC	GO:00 98800	Inner mitochondrial membrane protein complex	64/1844	112/ 13385	4.42E-13	4.40E-11	Ndufb8/Chchd6/Ndufa8/Ndufb2/Ndufs6l1/Ndufb6/Timm22/Ndufv1/Uqcrq/Cyc1/Cox6a1/Atp5f1d/Smdt1/ Ndufc1/Mt-co3/Uqcrc1/Ndufs7/Cox7a2/Timm17a/Atp5me/Apoo/Atp5mg/Ndufc2/Chchd10/Uqcrh/Cox7c/Ndufa4/Ndufv3/ A tp5f1b/Cox7a2l/Cox4i1/Atp5pb/Timm21/Timm10/Pam16/Uqcr10/Cox5b/Ndufa12/Atp5mk/Atp5po/ Ndufa3-ps3/Ndufs4/Phb2/Ndufa6/Ndufb5/Ndufa3/Timm23/Cox5a/Atp5pf/Dmac2/Ndufa11/Ndufab1/Atp5f1e/Atp5pd/ Ndufb10/Chchd3/Mt-co2/Uqcrb/Ndufs3/Trmt10b/Ndufs8/Atp5mk-ps2/Mt-co1/Ndufa9	64
CC	GO:00 05761	Mitochondrial ribosome	48/1844	67/ 13385	4.60E-13	4.40E-11	M rpl28/M rpl 27/M rpl37/M rpl3/M rpl44/M rps14/M rps6/Mtg2/Chchd1/M rpl4/M rpl12/M rps26/M rpl55/M rps12/ Mpv17l 2/Mrpl 14/Mrpl21/Mrpl46/Mrpl18/Mrpl20/Mrpl35/Mrpl54/Mrpl17/Mrps18c/Mrpl2/Mrpl49/Mrps18a/Mrps16/ Mrpl41/Mrps10/Mrps27/Mrps7/mrpl24/Mrpl48/Mrpl15/Mrpl19/Mrpl58/Mrpl13/Mrps15/mrpl9/Mrps21/ Mrps 18b/mrpl11/ENSRNOG00000068816/Mrpl16/Mrpl57/Mrpl43/Mrpl36	48
CC	GO:00 00313	ORganellar ribosome	48/1844	67/ 13385	4.60E-13	4.40E-11	Mrpl28/Mrpl 27/Mrpl37/Mrpl3/Mrpl44/Mrps14/Mrps6/Mtg2/Chchd1/Mrpl4/Mrpl12/Mrps26/Mrpl55/Mrps12/ Mpv17l 2/Mrpl 14/Mrpl21/Mrpl46/Mrpl18/Mrpl20/Mrpl35/Mrpl54/Mrpl17/Mrps18c/Mrpl2/Mrpl49/Mrps18a/Mrps16/ Mrpl41/Mrps10/Mrps27/Mrps7/mrpl24/Mrpl48/Mrpl15/Mrpl19/Mrpl58/Mrpl13/Mrps15/mrpl9/Mrps21/ Mrps 18b/mrpl11/ENSRNOG00000068816/Mrpl16/Mrpl57/Mrpl43/Mrpl36	48
CC	GO:00 70469	Respiratory chain	47/1844	75/ 13385	1.25E-08	5.95E-07	Ndufb8/Ndufa8/Ndufb2/Ndufs6l1/Ndufb6/Rab5if/Ndufv1/Uqcrq/Cyc1/Cox6a1/Ndufc1/ Mt-co3/Uqcrc1/Ndufs7/Cox7a2/Ndufc2/Uqcrh/Cox7c/Ndufa4/Ndufv3/Cox7a2l/Cox4i1/Uqcr10/Cox6b1/Cox5b/Ndufa 12/Sdhc/Ndufa3-ps3/Mt-cyb/Ndufs4/Ndufa6/Ndufb5/Ndufa3/Sdhd/Cox5a/Dmac2/Ndufa11/Ndufab1/Ndufb10/Mt- co2/Cox6b1/Uqcrb/Coa6/Ndufs3/Ndufs8/Mt-co1/Ndufa9	47
CC	GO:00 98803	Respiratory chain complex	46/1844	71/ 13385	4.47E-09	2.33E-09	Ndufb8/Ndufa8/Ndufb2/Ndufs6l1/Ndufb6/Ndufv1/Uqcrq/Cyc1/Cox6a1/Ndufc1/Mt- co3/Uqcrc1/Ndufs7/Cox7a2/Ndufc2/Uqcrh/Cox7c/Ndufa4/Ndufv3/Cox7a2l/Cox4i1/Uqcr10/Cox6b1/Cox5b/Nduf a 12/Sdhc/Ndufa3-ps3/Mt-cyb/Ndufs4/Ndufa6/Ndufb5/Ndufa3/Sdhd/Cox5a/Dmac2/Ndufa11/Ndufab1/Ndufb10/Mt- co2/Cox6b1/Uqcrb/Coa6/Ndufs3/Ndufs8/Mt-co1/Ndufa9	46

Cat.: Category; Descrip.: description; #: counts. Note: Category: Classification of GO databases, including biological processes (BP), cellularcomponents(CC), molecularfunction(MF).GOID: Unique identification id of Gene Ontology database. Description: Function description correspondingtothe GO number. GeneRatio: Ratio between the numberof differentially expressed genes in each GO term and all differentially expressed genes that can be found in GO database. BgRatio: In background GO database, the ratio of all genes concerning this GO term to all genes. *P* value: Statistics category term; abbreviation for probability value. *P*_adj_: Adjusted p-value. Generally, GO Terms with Corrected_*P* value <0.05 are significant enrichment. geneName: Differentially expressed genes in this term. Count: The numberof difference gene annotated to GO number.

**Figure 7:**
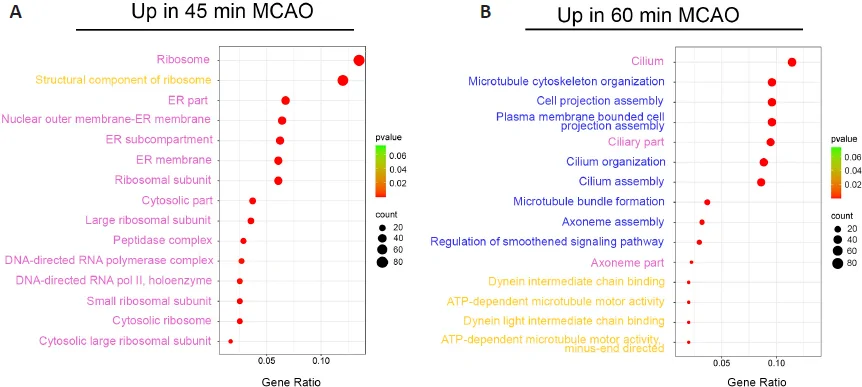
Gene Ontology (GO) enrichment analysis for the upregulated pathways in the cerebral ipsilateral cortex comparing the mild (A) and moderate ischemia rats (B) after 15 days of reperfusion. The y-axis shows the 15 most significant GO annotations (blue for biological processes, orange for molecular functions, and purple for cellular compartments). The x-axis indicates the ratio of the number of differentially expressed genes linked with the GO term to the total number of differentially expressed genes. The point size represents the number of genes annotated to a specific GO term, and the point color indicates the adjusted *P*-value. ER: Endoplasmic reticulum; MCAO: middle cerebral artery occlusion; Pol: polymerase.

When comparing the upregulated GO terms between mild and moderate ischemia, GO analysis revealed significant differences in cytosolic ribosomes and endoplasmic reticulum function (**Figure 7A** and **Additional Table 5**). Analysis of the downregulated GO terms showed a significant decrease in microtubule-related cell functions (**Figure 7B** and **Additional Table 6**).

**Additional Table 5:** Gene Ontology (GO) enrichment analysis for upregulated pathways in the cerebral ipsilateral cortex after 15 days of reperfusion in mild ischemia rats compared with moderate ischemia rats

Cat.	GOID	Descrip.	Gene ratio	Bg ratio	*P* value	*P*_adj_	geneName	#
CC	GO:00 05840	Ribosome	80/593	492/1366 6	2.10E-11	9.72E-09	LOC102555453/AABR07029195.1/ENSRNOG00000068920/Zcchc17/AC135389.1/Rsl24d1/Oscp1/Mrps18c/ Rps27a-ps1/Rps20-ps10/LOC100365839/Rpl39-ps13/Rps2-ps 6/RGD1564606/Rpl30l2/Rpl31/ LOC100361933/LOC686074/ENSRNOG00000063885/Mrpl36/Mrps14/AABR07049516.1/Rps 3a/Rplp1/Rps13/Rpl22l1/Rps14/Chchd1/LOC108348870/Rpl22/LOC498555/Rpl15/Rps7/Rpsa/Rpl18 /Mpv17l2/Pelo/ENSRNOG00000063418/LOC100360573/Pigu/Rpl23/Mrpl55/Rpl9-ps30/Rps27a-ps 2/Rpl19/ Rps4x/Rpl26/LOC687780/Rps24/Rps3/Rps13/Rps15/Rack1/Rpl36a/Rps25/Rpl35/Mrpl12/Rpl32/Rps19 /Rpl 11/Rps23/Mrpl3/Rps4y2/Rpl27/Rpl13/Rpl10a/LOC100363452/Rpsa-ps4/LOC120094951/ Rpl18a/Mrpl21/Rpl7a/Rps15a/Mrpl54/LOC100359505/Rps5/Rpl17/Rpl8-ps7/Rpl34-ps1/RGD1562402	80
MF	GO:00 03735	Structural constituent of ribosome	72/600	450/1422 1	3.73E-09	2.06E-06	LOC102555453/AABR07029195.1/ENSRNOG00000068920/AC135389.1/Rsl24d1/Oscp1/Mrps18c/Rps27a-ps 1/Rps20- ps10/LOC100365839/Rpl39-ps13/Rps2-ps 6/RGD1564606/Rpl30l2/Rpl31/LOC100361933/LOC686074/ ENSRNOG00000063885/Mrpl36/Mrps14/AABR07049516.1/Rps 3a/Rplp1/Mrps34/Rps13/Rpl22l1/Rps14/ LOC108348870/Rpl22/LOC498555/Rpl15/Rps7/Rpsa/Rpl18/ENSRNOG00000063418/LOC100360573/Rpl23/Mrpl55/ Rpl9-ps30/Rps27a-ps 2/Rpl19/Rps4x/Rpl26/LOC687780/Rps24/Rps3/Rps13/Rps15/Rpl36a/Rps25/Rpl35/Mrpl12/Rpl32/Rps19/Rpl11 /Rps 23/Mrpl3/Rps4y2/Rpl27/Rpl13/Rpl10a/LOC100363452/Rpsa-ps4/LOC120094951/Rpl18a/Rps15a/LOC100359505/ Rps5/Rpl17/Rpl8-ps7/Rpl34-ps1/RGD1562402	72
CC	GO:00 44432	Endoplasmic reticulum part	40/593	429/1366 6	4.28E+08	0.0001652 61255129 481	Saraf/Stard3/Rpn1/Sec62/Hsd17b12/Calr/Sec61b/Slc35b1/Rdh11/Tor1a/Hsp90b1/Emd/Ddost/Erp44/Vapa/ Bloc1s1/C2h4orf3/Hspa5/Derl2/Sdf2l1/Kdelr2/Spcs1/Bcap31/Tmem41b/Spcs2/Selenof/Tmed2/Pigu/Selenok/Dad1/ Ostc/Cept1/Hm13/Emc6/Jagn1/Spcs3/Aup1/Tram1/Abhd12/Clptm1l	40
CC	GO:00 42175	Nuclear outer membrane- endoplasmic reticulum membrane network	38/593	378/1366 6	1.24E+08	6.37E+09	Saraf/Stard3/Cnep1r1/Rpn1/Sec62/Hsd17b12/Calr/Sec61b/Slc35b1/Rdh11/Tor1a/Hsp90b1/Emd/Ddost/Erp44/ Vapa/Bloc1s1/C2h4orf3/Hspa5/Derl2/Kdelr2/Spcs1/Tmem41b/Spcs2/Tmed2/Pigu/Selenok/Dad1/Ostc/Cept1/ Hm13/Emc6/Jagn1/Spcs3/Aup1/Tram1/Abhd12/Clptm1l	38
CC	GO:00 98827	Endoplasmic reticulum subcompartment	37/593	371/1366 6	2.07E+08	9.59E+08	Saraf/Stard3/Rpn1/Sec62/Hsd17b12/Calr/Sec61b/Slc35b1/Rdh11/Tor1a/Hsp90b1/Emd/Ddost/Erp44/Vapa/ Bloc1s 1/C2h4orf3/Hspa5/Derl2/Kdelr2/Spcs1/Tmem41b/Spcs2/Tmed2/Pigu/Selenok/Dad1/Ostc/Cept1/Hm13/Emc6 /Jagn1/Spcs3/Aup1/Tram1/Abhd12/Clptm1l	37
CC	GO:00 05789	Endoplasmic reticulum membrane	36/593	362/1366 6	3.04E+08	0.0001280 03944408 76	Saraf/Stard3/Rpn1/Sec62/Hsd17b12/Calr/Sec61b/Slc35b1/Rdh11/Tor1a/Hsp90b1/Ddost/Erp44/Vapa/Bloc1s1/ C2h4orf3/Hspa5/Derl2/Kdelr2/Spcs1/Tmem41b/Spcs2/Tmed2/Pigu/Selenok/Dad1/Ostc/Cept1/Hm13/Emc6/Jagn1/ Spcs3/Aup1/Tram1/Abhd12/Clptm1l	36
CC	GO:00 44391	Ribosomal subunit	36/593	153/1366 6	3.65E-04	8.46E-01	AABR07029195.1/Zcchc17/Mrps 18c/Rps20-ps10/Rps2-ps6/Rpl30l2/LOC686074/Mrpl36/Mrps14/LOC498555/Rpl15/Rpsa/Mpv17l 2/Mrpl55/Rpl19/Rps4x/Rpl26/Rps24/Rps3/Rps15/Rack1/Rps25/Rpl35/Mrpl12/Rps23/Mrpl3/Rpl27/Rpl10a/ Rpsa-ps4/Mrpl21/Rpl7a/Mrpl54/Rps5/Rpl17/Rpl8-ps7/RGD1562402	36
CC	GO:00 44445	Cytosolic part	22/593	148/1366 6	4.06E+07	2.69E+08	Zcchc17/Rpl30l2/LOC686074/Ciao2a/Bloc1s3/Bloc1s1/Borcs7/LOC498555/Rpl15/Pelo/Arl6ip5/Rpl19/Rps4x/Rpl2 6/Rps 24/Rps15/Rps25/Rpl35/Ciao2b/Rpl27/Rpl7a/Cct5	22
CC	GO:00 15934	Large ribosomal subunit	21/593	94/13666	4.18E+03	6.45E+06	Zcchc17/Rpl30l2/LOC686074/Mrpl36/LOC498555/Rpl15/Mpv17l2/Mrpl55/Rpl19/Rpl26/Rpl35/Mrpl12/Mrpl3/ Rpl 27/Rpl10a/Mrpl21/Rpl7a/Mrpl54/Rpl17/Rpl8-ps7/RGD1562402	21
CC	GO:19 05368	Peptidase complex	17/593	96/13666	6.95E+07	4.02E+09	Psma 3/Fcnb/Psma5/Psma1/AABR07040855.1/Spcs1/Sgf29/Taf10/Spcs2/Psmc2/Psmb1/Psmb4/Sem1/Spcs3/Tad a1/Taf9/Psma4	17
CC	GO:00 16591	DNA-directed RNA polymerase II, holoenzyme	15/593	95/13666	1.35E+09	0.0004794 99991003 457	Ints12/Gtf2e2/Med29/Med10/RGD1562272/Med21/Polr2d/Taf10/Taf13/Med28/Med30/Sem1/Taf9/Med9/Gtf2 h5	15
CC	GO:00 00428	DNA-directed RNA polymerase complex	16/593	111/1366 6	2.28E+09	0.0007040 96896450 118	Ints12/Gtf2e2/Med29/Med10/Polr1d/RGD1562272/Med21/Polr2d/Taf10/Taf13/Med28/Med30/Sem1/Taf9/Med9/Gtf2h5	16
CC	GO:00 15935	Small ribosomal subunit	15/593	60/13666	2.65E+06	2.45E+08	AABR07029195.1/Mrps 18c/Rps20-ps10/Rps2-ps6/Mrps14/Rpsa/Rps4x/Rps24/Rps3/Rps15/Rack1/Rps25/Rps23/Rpsa-ps4/Rps5	15
CC	GO:00 22626	Cytosolic ribosome	15/593	47/13666	6.32E+04	7.32E+06	Zcchc17/Rpl 30l2/LOC686074/LOC498555/Rpl15/Pelo/Rpl19/Rps4x/Rpl26/Rps24/Rps15/Rps25/Rpl35/Rpl27/Rpl7a	15
CC	GO:00 22625	Cytosolic large ribosomal subunit	10/593	27/13666	9.48E+06	7.32E+08	Zcchc17/Rpl 30l2/LOC686074/LOC498555/Rpl15/Rpl19/Rpl26/Rpl35/Rpl27/Rpl7a	10

Cat.: Category; Descrip.: description; #: counts. Note: Category: Classification of GO databases, including biological processes (BP), cellularcomponents(CC), molecularfunction(MF).GOID: Unique identification id of Gene Ontology database. Description: Function description correspondingtothe GO number. GeneRatio: Ratio between the numberof differentially expressed genes in each GO term and all differentially expressed genes that can be found in GO database. BgRatio: In background GO database, the ratio of all genes concerning this GO term to all genes. *P* value: Statistics category term; abbreviation for probability value. *P*_adj_: Adjusted p-value. Generally, GO Terms with Corrected_*P* value <0.05 are significant enrichment. geneName: Differentially expressed genes in this term. Count: The numberof difference gene annotated to GO number.

**Additional Table 6:** Gene Ontology (GO) enrichment analysis for downregulated pathways in the cerebral ipsilateral cortex after 15 days of reperfusion in mild ischemia rats compared with moderate ischemia rats

Cat.	GOID	Descrip.	Gene ratio	Bg ratio	*P* value	*P*_adj_	geneName	#
CC	GO:00 05929	Cilium	41/359	453/ 13666	3.84E+02	1.42E+05	Dnah9/Nphp3/Spag17/Pkhd1/Dnah7/Dnah2/Ccdc120/Cc2d2a/Spata6l/Hydin/Kif27/Dlg5/Crocc/Cplane2/Dnah5/ Ush2a/C2cd3/Ift172/Cfap65/Dnah1/Drc1/Nin/Cep162/Traf3ip1/Intu/Gli3/Npr2/Rpgrip1l/Eno4/Dync2h1/Odf2/ P kd1/Strc/Wdr19/Bbs7/Ppp1r32/Glis2/Fank1/Alpk1/Mapk15/Cep131	41
BP	GO:00 00226	Microtubule cytoskeleton organization	39/407	433/ 14869	5.98E+03	3.66E+06	Deup1/Spag17/Pkhd1/Dnah7/Dnah2/Ccdc120/Cc2d2a/Hydin/Cfap44/Crocc/Cfap46/Cplane2/Pcnt/Ccdc187/ Hoo k2/Dnah5/C2cd3/Numa1/Ift172/Cfap65/Cep70/Dclk2/Drc1/Nin/Cep120/Traf3ip1/Rock1/Limk2/Nckap5l/Spice1/ Odf2/Pkd1/Cu l9/Cep350/Mapk15/Cep131/Dcx/Cu l7/Haus6	39
BP	GO:00 30031	Cell projection assembly	39/407	399/ 14869	4.92E+02	3.76E+05	Nphp3/Spag17/Pkhd1/Def8/Dnah7/Dnah2/Cc2d2a/Ca rmil1/Hydin/Cfap44/Kif27/Crocc/Cfap46/Cplane2/Pcnt/ Dnah5/C2cd3/Ift172/Cfap65/Cep70/LOC690035/Drc1/Cep162/Cep120/Rock1/Intu/Rpgrip1l/Limk2/Dync2h1/Epha2 /Odf2/Cep350/Wdr19/Bbs7/Nek1/Alpk1/Mapk15/Cep131/Dcx	39
BP	GO:01 20031	Plasma membrane bounded cell projection assembly	39/407	396/ 14869	3.89E+01	3.76E+05	Nphp3/Spag17/Pkhd1/Def8/Dnah7/Dnah2/Cc2d2a/Carmil1/Hydin/Cfap44/Kif27/Crocc/Cfap46/Cplane2/Pcnt/ Dn ah5/C2cd3/Ift172/Cfap65/Cep70/LOC690035/Drc1/Cep162/Cep120/Rock1/Intu/Rpgrip1l/Limk2/Dync2h1/Epha2 /Odf2/Cep350/Wdr19/Bbs7/Nek1/Alpk1/Mapk15/Cep131/Dcx	39
CC	GO:00 44441	Ciliarypart	34/359	352/ 13666	5.18E+02	9.55E+04	Dnah9/Nphp3/Spag17/Pkhd1/Dnah7/Dnah2/Ccdc120/Cc2d2a/Spata6l/Hydin/Dlg5/Crocc/Cplane2/Dnah5/Ush2a /C2cd3/Ift172/Cfap65/Drc1/Nin/Cep162/Traf3ip1/Intu/Gli3/Rpgrip1l/Eno4/Dync2h1/Odf2/Wdr19/Bbs7/ Ppp1r32/Fank1/Mapk15/Cep131	34
BP	GO:00 44782	Cilium organization	36/407	287/ 14869	1.93E+00	2.95E+03	Nphp3/Spag17/Pkhd1/Dnah7/Dnah2/Cc2d2a/Hydin/Cfap44/Kif27/Crocc/Cfap46/Cplane2/Pcnt/Dnah5/C2cd3/ Ift172/Cfap65/Cep70/LOC690035/Drc1/Cep162/Cep120/Intu/Rpgrip1l/Limk2/Eno4/Dync2h1/Odf2/Cep350/Wdr19 /Bbs 7/N ek1/Alpk1/Mapk15/Cep131/Dcx	36
BP	GO:00 60271	Cilium assembly	35/407	265/ 14869	9.34E-01	2.86E+03	Nphp3/Spag17/Pkhd1/Dnah7/Dnah2/Cc2d2a/Hydin/Cfap44/Kif27/Crocc/Cfap46/Cplane2/Pcnt/Dnah5/C2cd3/ Ift172/Cfap65/Cep70/LOC690035/Drc1/Cep162/Cep120/Intu/Rpgrip1l/Limk2/Dync2h1/Odf2/Cep350/Wdr19/Bbs7 /Nek1/Alpk1/Mapk15/Cep131/Dcx	35
BP	GO:00 01578	Microtubule bundle formation	15/407	90/14869	1.97E+06	1.01E+09	Spag17/Dnah7/Dnah2/Cc2d2a/Hydin/Cfap44/Cfap46/Cplane2/Dnah5/Numa1/Cfap65/Drc1/Nckap5l/Cep131/Dcx	15
BP	GO:00 35082	Axoneme assembly	13/407	72/14869	6.55E+06	2.86E+09	Spag17/Dnah7/Dnah2/Cc2d2a/Hydin/Cfap44/Cfap46/Cplane2/Dnah5/Cfap65/Drc1/Cep131/Dcx	13
BP	GO:00 08589	Regulation of smoothened signaling pa thway	12/407	66/14869	1.95E+06	7.45E+09	Chrd/Dlg5/Rora/Cplane2/C2cd3/Ift172/Megf8/Intu/Gli3/Rpgrip1l/Dync2h1/Glis2	12
CC	GO:00 44447	Axoneme part	8/359	25/13666	1.54E+07	1.89E+09	Dnah9/Spag17/Dnah7/Dnah2/Hydin/Dnah5/Drc1/Cep162	8
MF	GO:00 45505	Dynein intermediate chain binding	8/406	23/14221	1.39E+07	1.70E+09	Dnah9/Dnah7/Dnah2/Dnah5/Dnah1/Dnah3/Dync2h1/Dnah6	8
MF	GO:19 90939	ATP- dependent microtubule motor activity	8/406	20/14221	3.84E+06	6.26E+08	Dnah9/Dnah7/Dnah2/Dnah5/Dnah1/Dnah3/Dync2h1/Dnah6	8
MF	GO:00 51959	Dynein light intermediate chain binding	8/406	18/14221	1.40E+06	3.43E+07	Dnah9/Dnah7/Dnah2/Dnah5/Dnah1/Dnah3/Dync2h1/Dnah6	8
MF	GO:00 08569	ATP- dependent microtubule motor activity, minus-end- directed	8/406	16/14221	4.34E+04	2.12E+08	Dnah9/Dnah7/Dnah2/Dnah5/Dnah1/Dnah3/Dync2h1/Dnah6	8

Cat.: Category; Descrip.: description; #: counts. Note: Category: Classification of GO databases, including biological processes (BP), cellularcomponents(CC), molecularfunction(MF).GOID: Unique identification id of Gene Ontology database. Description: Function description correspondingtothe GO number. GeneRatio: Ratio between the numberof differentially expressed genes in each GO term and all differentially expressed genes that can be found in GO database. BgRatio: In background GO database, the ratio of all genes concerning this GO term to all genes. *P* value: Statistics category term; abbreviation for probability value. *P*_adj_: Adjusted p-value. Generally, GO Terms with Corrected_*P* value <0.05 are significant enrichment. geneName: Differentially expressed genes in this term. Count: The numberof difference gene annotated to GO number.

These data reveal a severity-dependent gene expression landscape, in which moderate ischemia amplifies immune activation and metabolic disruption, while distinct ribosomal and cytoskeletal pathways differentiate mild from moderate injury.

### Quantitative reverse transcription-polymerase chain reaction validation of the RNA sequencing results

The following key inflammatory and ER-stress markers were tested by qPCR and western blot across mild and moderate MCAO models to validate the RNA-seq findings.

**Figure 8:**
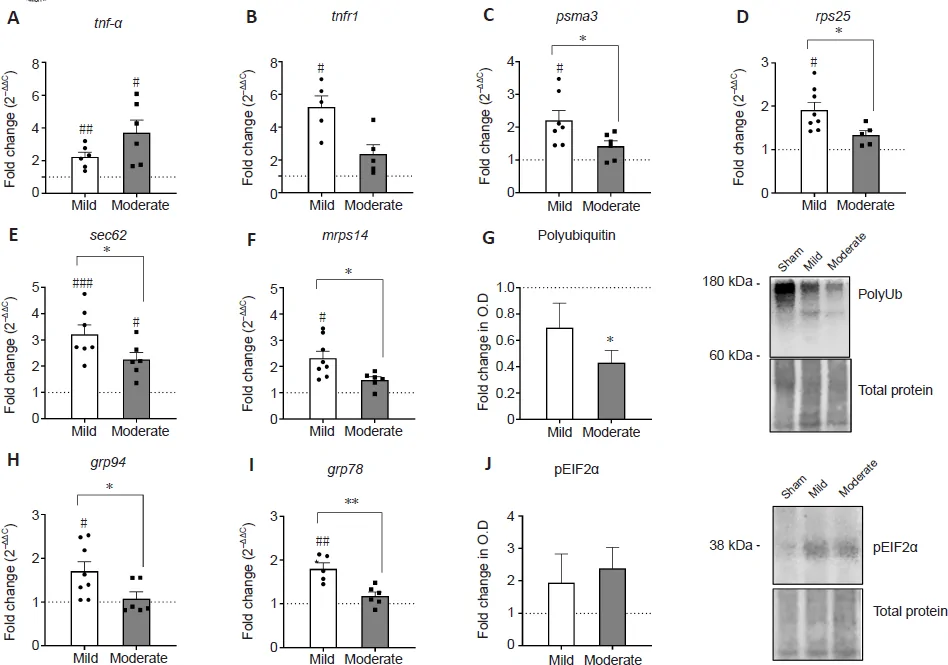
Validation of the RNA sequencing (RNA-seq) results. Changes between the mild and moderate ischemia rats after 15 days of reperfusion were validated by measuring the fold change (2^–ΔΔCt^) in the transcript levels of tumor necrosis factor (*tnf-α*) (A) and *tnfr1* (B), indicative of inflammation; *psma3* (C), indicative of proteasome activation; ribosomal protein eS25 (*rps25*; D) and *sec62* (E), indicative of protein synthesis in the cytosol and mitochondrial ribosomal protein S14 (*mrps14*) (F), indicative of protein synthesis in the mitochondria; *grp94* (H) and *grp7*8 (I), indicative of endoplasmic reticulum stress. The amount of polyubiquitinated protein (G) and phosphorylated EIF2α (J) were measured by western blotting to validate endoplasmic reticulum stress and protein synthesis, respectively. Representative images of the corresponding Western blot membranes are also included. Data are presented as the mean ± standard error of the mean (*n* ≥ 5). *P < 0.05, **P < 0.01; #*P* < 0.05, ##*P* < 0.01, ###*P* < 0.001, *vs.* sham (dotted line) (one-way analysis of variance followed by Tukey’s *post hoc* test).

The RNA-seq analysis indicated upregulation of inflammation-related genes in both mild and moderate ischemia compared with sham animals. qPCR indicated a significant increase in the transcript levels of the cytokine tumor necrosis factor (*Tnf-α*) and its main receptor *Tnfr1*, which confirms inflammatory activation in mild and moderate ischemia rats (**Figure 8A** and **B**).

We also measured the transcript levels of some representative genes that encode proteins involved in protein processing, namely proteasome subunit alpha 3 (*Psma3*); ribosomal protein eS25 (*Rps25*), a component of the 40S subunit; *Sec62*, a preprotein translocation factor; and mitochondrial ribosomal protein S14 (*Mrps14*). The transcripts were significantly upregulated in mild ischemia compared with moderate ischemia animals; of note, the moderate ischemia group showed transcript levels similar to the sham group (**Figure 8C–F**). Furthermore, western blotting showed significantly reduced ubiquitination in the moderate ischemia rats compared with the sham rats. Ubiquitination in the mild ischemia remained lower than in the sham group, but it was not significantly different compared with the moderate ischemia or sham rats (**Figure 8G**).

Finally, we assessed transcripts that encode crucial endoplasmic reticulum stress chaperones and polyubiquitinated proteins. Transcription of *Grp94* and *Grp78* was increased significantly in the ipsilateral cerebral cortex of mild ischemia rats after 15 days of reperfusion compared with the sham (*P* = 0.049 and *P* = 0.0003, respectively) and moderate ischemia animals (*P* = 0.047 and *P* = 0.001, respectively). The levels of these chaperone transcripts were similar in the sham and moderate ischemia groups (**Figure 8H** and **I**). Based on western blotting, although both mild and moderate ischemia increased phosphorylated EIF2α to a similar extent compared to the sham-operated animals, the differences were not statistically significant (**Figure 8J**).

These results confirm heightened inflammatory signaling in both injury severities, while only mild ischemia triggers sustained upregulation of protein-processing and ER stress pathways, indicating divergent responses according to ischemic severity.

## Discussion

We compared the evolution of the infarct volume, the neurological deficit, and gene expression between mild and moderate forms of MCAO to understand the progression of stroke in the midterm. After 48 hours of reperfusion, the moderate ischemia group showed a neurological deficit based on the mNSS (Senda et al., 2011). The findings confirm previous results of this moderate ischemia model regarding the behavioral test results and the infarct volume (Santos-Galdiano et al., 2018; Ugidos et al., 2022). In contrast, these tests failed to detect a cognitive deficit in the mild ischemia at this time, although the infarct volume was similar in both models. Based on the TTC assay, there was a greater loss of staining after the first days compared with longer periods of reperfusion, as surviving cells after an ischemic insult may not be active enough to be detected macroscopically, and transient impairment of dehydrogenase activity may result in negative TTC staining (Yoon et al., 2018). This may explain the discrepancy between the infarct volume and cognitive deficit during the first days of reperfusion.

We observed progressive amelioration of the neurological deficit in the mild and moderate ischemia groups along the 15 days of reperfusion, as evidenced by the cylinder, adhesive, and apomorphine-induced rotation tests. Previous studies using the cylinder test have described the presence of forelimb asymmetry after ischemic injury to the sensorimotor cortex (Grow et al., 2003; Roome and Vanderluit, 2015) and striatal damage (Hobbs and Oorschot, 2008; Cameron et al., 2015). We noted forelimb asymmetry in the mild and moderate ischemia rats after 7 days of reperfusion, although the effect was stronger in the moderate ischemia rats. The results of this assay also support the lack of recovery after 15 days of reperfusion and its usefulness in evaluating putative drugs against stroke in both models.

The adhesive removal test has been used in rodents to assess motor coordination and sensory neglect in different models of brain injury, including stroke (Balkaya et al., 2013a; Rewell et al., 2017; Fanaei et al., 2024). This test has been reported to be useful in rats to assess changes up to 4 weeks after stroke and to differentiate between sensory and motor function (Trueman et al., 2017; Yilmaz et al., 2023; Mehta et al., 2024). Based on the adhesive removal test results, the moderate ischemia group showed greater sensory than motor deficits during reperfusion compared with the mild ischemia group. In both models, sensory and motor deficits appear to be attenuated after 15 days of reperfusion, which supports the utility of this test when assessing the short-term and medium-term effects of MCAO.

The wire hanging test has been used in rodent models to evaluate motor deficits and is based on the latency of the rat to fall after being suspended from an elevated wire. Furthermore, it has been included in the Garcia index, which is designed to assess motor and sensory deficits following MCAO (Chang et al., 2019; Owjfard et al., 2024). In the present study, this test did not reveal differences between mild and moderate ischemia experimental groups at the times we analyzed.

The apomorphine-induced rotation test is a well-standardized assay to evaluate the integrity of the dopaminergic nigro-striatal projections in animal models of Parkinson’s disease (Jiang et al., 2022; Ren et al., 2023; Ortirz-Islas et al., 2024). The ipsilateral damage, due to the absence of dopamine (DA) stimulation, has been assessed using pharmacological agents such as amphetamine, cocaine, and apomorphine that promote the release of dopamine or inhibit its reuptake, resulting in a contralateral circling behavior (Balkaya et al., 2013a, b, 2018; Björklund and Dunnett, 2019). Apomorphine is a DA receptor agonist that acts postsynaptically. Hyperstimulation of supersensitive DA receptors in the denervated striatum induces rotation in the contralateral direction. This test indirectly measures the loss or recovery of striatal DA neurotransmission. In general, a response of over 300 rotations per hour may indicate a maximum of 90% loss of DA neurons in the substantia nigra (Hsieh et al., 2011). This test has also been employed to assess the effects of ipsilateral MCAO, yielding robust and applicable results for MCAO studies (Trueman et al., 2017; Balkaya et al., 2013a, b, 2018; Noh et al., 2020). We found that this test could discriminate between the mild and moderate ischemia rats at both 7 and 15 days of reperfusion, indicating that impairments in the dopaminergic system are dynamic and dependent on both the duration of ischemia and the reperfusion.

Overall, the behavioral tests we performed revealed that the mild and moderate ischemia rats present motor and sensory differences, which could be used to assess putative anti-stroke agents in the midterm. We conducted RNA-seq of the penumbra region after 15 days of reperfusion to identify the relevant molecular pathways underlying these differences. There were significant discrepancies in the pathophysiological conditions of the mild and moderate ischemia animals, mirrored by the large number of up- and downregulated genes in the mild and moderate ischemia compared with the sham animals, which remained when we compared the moderate and mild models between them. The differences in the total number of differentially expressed genes indicate that a slight (15-minute) variation in the ischemia time, despite only leading to minor differences in behavior and the infarct volume, could lead to a completely different molecular scenario, a factor that is sometimes overlooked.

In the mild and moderate ischemia, inflammation-related processes were the top hits compared with the sham group. After 15 days of reperfusion, transcripts related to the inflammatory response remained upregulated, indicating that this process is a major therapeutic target to ameliorate stroke progression (Candelario-Jalil et al., 2022; Kumari et al., 2024; Zhao et al., 2024). qRT-PCR validated the RNA-seq results, with *tnf-α* and *tnfr1* overexpression in mild and moderate ischemia. However, the *tnf-α*/*tnfr1* ratio was different between the mild and moderate ischemia groups. Because TNFR1 activation can subsequently activate several pathways, such as apoptosis, necroptosis, or nuclear factor kappa B (*nf-kb*) (Li et al., 2025), our results suggest that inflammation could progress differently in mild and moderate ischemia.

Our GO analysis indicated that the mild ischemia group presented higher protein synthesis than the moderate ischemia group. We validated this finding by examining the expression of key cytosolic and mitochondrial ribosome subunits involved in protein synthesis. The transcripts of *rps25*, which encode the 40S ribosomal protein S25 (Tutak et al., 2025), and *mrps14*, which encodes the mitochondrial ribosomal protein S14 (Tasneem et al., 2024), were increased, supporting increased cytosolic and mitochondrial protein translation in the mild ischemia model compared with the moderate ischemia model. This idea is also supported by higher levels of the endoplasmic reticulum translocator complex *sec62* (Jung and Kim, 2021), despite the existence of ischemia-induced ER stress after 15 days of reperfusion, supported by the levels of *grp78* and *grp94* transcripts and phosphorylated EIF2α. Given that phosphorylated EIF2α inhibits translation (Gordiyenko et al., 2019) and there were similar levels of phosphorylated EIF2α in the mild and moderate ischemia, protein translation blockade induced by endoplasmic reticulum stress is likely not the mechanism that underlies the differences in the protein synthesis between the mild and moderate ischemia groups. We observed low levels of polyubiquitinated proteins in the mild and moderate ischemia animals compared with the sham animals. An adequate balance of degradative and non-degradative ubiquitin-ligase systems has been related to neuronal viability (Schmidt et al., 2021). We hypothesize that the lower ubiquitin levels in the mild and moderate ischemia groups compared with the sham group mirror the tissue damage and correlate with the neurological deficit observed in these models. Taken together, these data suggest increased protein synthesis after 15 days of reperfusion in mild ischemia, which probably represents an attempt to recover the tissue. Presynaptic protein synthesis plays a crucial role in brain plasticity (Perrone-Capano et al., 2021). Thus, the increased protein synthesis observed in mild ischemia may explain the improved tissue recovery, neuronal plasticity, and better performance in behavioral assays noted in our study. The clinical relevance of these results is substantial. Findings from MCAO models in rats guide clinical approaches to stroke by emphasizing the need for rapid intervention to reduce ischemic duration. Our results highlight the relationship between infarct size and functional outcomes, mirroring human stroke progression. This kind of study provides further support for the development of personalized stroke management and neuroprotective therapies.

## Limitations

The study presents several limitations that should be noted. Only two discrete ischemia durations (45 and 60 minutes) were tested, which may obscure more nuanced thresholds of injury that could be explored in future research. Furthermore, the study was confined to a single rodent strain and sex, potentially limiting generalizability. Incorporating both sexes and multiple strains in subsequent studies would help clarify the influence of genetic and hormonal factors on ischemic thresholds.

Regarding the transcriptomic profiling, the use of bulk tissue masks cell type–specific responses; single-cell or spatial transcriptomics could help resolve the contributions of neurons, glia, and infiltrating immune cells. Finally, outcomes were assessed only up to 15 days post-reperfusion, leaving longer-term recovery and plasticity unexplored. Extended time-course studies are therefore warranted to determine the persistence or resolution of inflammatory and proteostatic alterations. Addressing these points will strengthen the translational relevance of our model and support optimized screening of neuroprotective agents.

## Conclusions

Differences in behavior and gene expression between mild and moderate ischemia suggest the existence of a threshold for the ischemia time (reached with moderate ischemia) from which motor and sensory behaviors and the ability to synthesize protein appear substantially impaired after 15 days of reperfusion. Our results also reveal chronic inflammation in mild and moderate ischemia and the presence of higher endoplasmic reticulum stress in mild compared with moderate ischemia. Our findings indicate that ischemia duration should be finely tuned based on the specific molecular processes targeted by the candidate drug, enhancing the precision and effectiveness of drug discovery in stroke.

## Additional files:

***Additional Table 1:*** *Gene Ontology (GO) enrichment analysis for upregulated pathways in the cerebral ipsilateral cortex after 15 days of reperfusion in mild ischemia rats compared with sham rats.*

***Additional Table 2:*** *Gene Ontology (GO) enrichment analysis for downregulated pathways in the cerebral ipsilateral cortex after 15 days of reperfusion in mild ischemia rats compared with sham rats.*

***Additional Table 3:*** *Gene Ontology (GO) enrichment analysis for upregulated pathways in the cerebral ipsilateral cortex after 15 days of reperfusion in moderate ischemia rats compared with sham rats.*

***Additional Table 4:*** *Gene Ontology (GO) enrichment analysis for downregulated pathways in the cerebral ipsilateral cortex after 15 days of reperfusion in moderate ischemia rats compared with sham rats.*

***Additional Table 5:*** *Gene Ontology (GO) enrichment analysis for upregulated pathways in the cerebral ipsilateral cortex after 15 days of reperfusion in mild ischemia rats compared with moderate ischemia rats.*

***Additional Table 6:*** *Gene Ontology (GO) enrichment analysis for downregulated pathways in the cerebral ipsilateral cortex after 15 days of reperfusion in mild ischemia rats compared with moderate ischemia rats.*

## Data Availability

*All relevant data are within the paper and its Additional files.*
